# Amino acid compound-specific isotope analysis reveals island mass effect subsidies in reef-associated Hawaiian zooplankton

**DOI:** 10.7717/peerj.21076

**Published:** 2026-04-29

**Authors:** Blake Stoner-Osborne, Rita García-Seoane, Christopher B. Wall, Natalie Wallsgrove, Jeffrey C. Drazen, Brian N. Popp

**Affiliations:** 1 Department of Oceanography, University of Hawaii at Manoa, Honolulu, HI, United States of America; 2 Department of Earth Sciences, University of Hawaii at Manoa, Honolulu, HI, United States of America; 3 Centro Oceanográfico de A Coruña, Instituto Español de Oceanografía (IEO-CSIC), A Coruña, Galicia, Spain

**Keywords:** Hawaiian Islands, Essential amino acids, Source amino acids, Trophic position, Food web connectivity, Pelagic consumers, North Pacific Subtropical Gyre

## Abstract

The Island Mass Effect (IME) is the nearshore enhancement of primary productivity around islands and atolls relative to offshore waters. Although its physical and biogeochemical drivers are well characterized, the IME’s influence on the diets and distributions of consumers remains poorly resolved. We applied amino acid compound-specific stable isotope analysis (AA-CSIA) to Hawaiian zooplankton sampled across nearshore–offshore and surface–deep gradients to test whether island-derived production alters isotopic composition and trophic structure in reef-associated assemblages relative to offshore counterparts across sites, seasons, and years. Essential amino acid δ^13^C values (δ^13^C_EAA_) normalized to their mean values displayed contrasting nearshore–offshore patterns: lysine and threonine δ^13^C values increased with distance from shore, whereas phenylalanine and valine values decreased. These patterns likely reflect shifts in zooplankton diet and the amino acid biosynthetic pathways of their primary producer prey along the coastal–oceanic gradient. Source amino acid δ^15^N values (δ^15^N_SAA_) declined offshore for lysine and phenylalanine but increased with depth, indicating spatial variation in nitrogen sources and greater reliance on microbially reworked organic matter at depth. Trophic position estimates based on δ^15^N values of glutamic acid and alanine relative to phenylalanine increased offshore and with depth, consistent with longer food webs and additional microzooplankton trophic steps in offshore waters. Multivariate analysis integrating δ^13^C_EAA_ and δ^15^N_SAA_ values clearly distinguished reef, offshore surface, and offshore deep zooplankton assemblages, revealing a conservative isotopic tracer of island-derived production in reef communities. These results demonstrate AA-CSIA’s utility for tracing island-derived productivity to consumers and clarifying biogeochemical connectivity between coastal and open-ocean food webs.

## Introduction

There are roughly 18,000 oceanic islands greater than one km^2^ worldwide, many of which lie within the Pacific basin (Weigelt, Jetz & Kreft, 2013). Nearshore waters in these regions often exhibit striking increases in primary production (up to two orders of magnitude) and in chlorophyll standing stocks (up to 86%) relative to adjacent offshore waters (Doty & Oguri, 1956; Gilmartin & Revelante, 1974; Gove et al., 2016). This phenomenon, called the “Island Mass Effect” (IME), arises from biogeochemical and biophysical processes (*e.g.*, upwelling, eddy activity, internal waves, runoff, submarine groundwater discharge) that locally enhance nutrient availability and stimulate near-island phytoplankton growth (Gove et al., 2016). The IME is widespread, having been documented around 91% of coral reef islands and atolls across the tropical Pacific, though its magnitude varies widely among and seasonally within island systems (Gove et al., 2016).

Across the tropical Pacific, zooplankton and micronekton communities may show pronounced nearshore–offshore structuring aligned with elevated productivity near islands. Enhanced phytoplankton biomass and nutrient supply within IME zones are thought to fuel higher abundances of zooplankton, micronekton, and other pelagic predators (Nadon et al., 2012; Williams et al., 2015). Similar bottom-up responses are hypothesized at shallow seamounts, where elevated chlorophyll concentrations mark localized productivity hotspots with increased catch rates of higher trophic level organisms (Leitner, Neuheimer & Drazen, 2020). However, aggregations of mid- to high-trophic-level organisms can also emerge from primarily physical mechanisms, including current entrainment and disrupted diel vertical migration (Boehlert & Mundy, 1993; Cascão et al., 2017; Rogers, 2018). These processes make it difficult to determine whether elevated consumer densities near islands reflect true biological subsidies from island-derived primary production, physical forcing, or some combination of both.

In the Hawaiian Islands, phytoplankton IME dynamics are well studied but their impacts on higher trophic level organisms remain poorly constrained (Doty & Oguri, 1956; Gilmartin & Revelante, 1974; Comfort et al., 2024). Zooplankton assemblages in Kāneʻohe Bay differ sharply from those offshore (Hirota & Szyper, 1976). Likewise, micronekton biomass and community composition shift markedly across nearshore–offshore gradients off the west coast of Hawaiʻi Island (Drazen et al., 2023). Both zooplankton and a distinct assemblage of micronekton (called the mesopelagic boundary layer) migrate toward shallow island slopes at night, presumably to exploit elevated prey availability in nearshore waters (Reid et al., 1991; Benoit-Bird & Au, 2006; Benoit-Bird, Zirbel & McManus, 2008). IME-driven nutrient gradients that increase primary productivity in nearshore waters may underlie nearshore–offshore differences in consumer community composition and biomass, however a lack of robust trophic tracers for IME-derived production has left this hypothesized mechanism untested. Establishing such tracers is crucial for determining whether, and over what spatial scales, enhanced nearshore productivity propagates through pelagic food webs and shapes zooplankton and micronekton community structure across coastal–open ocean gradients.

Amino acid compound-specific stable isotope analysis (AA-CSIA) offers a promising approach for developing a robust trophic tracer of island-derived production. Traditional bulk tissue or whole animal δ^13^C and δ^15^N analyses provide valuable information on food web structure and sources (Peterson & Fry, 1987) but are confounded by spatial and temporal baseline variability (Post, 2002). In contrast, AA-CSIA separates baseline and trophic effects by analyzing isotopic patterns among individual amino acids (McClelland & Montoya, 2002; Chikaraishi et al., 2007; Popp et al., 2007), thereby providing a more reliable measure of trophic dynamics. Source amino acids (SAAs) such as phenylalanine and lysine retain baseline δ^15^N values, whereas trophic amino acids (TAAs) such as glutamic acid and alanine δ^15^N values increase by ∼4–8‰ per trophic step, enabling precise trophic position (TP) estimates (Chikaraishi et al., 2009; Décima et al., 2017). Similarly, δ^13^C values of essential amino acids (δ^13^C_EAA_) trace primary producer carbon sources through food webs because animals cannot synthesize EAAs *de novo*. Instead, they assimilate EAAs directly from their diet with minimal isotopic fractionation (Fantle et al., 1999; Manlick & Newsome, 2022). Distinct δ^13^C_EAA_ profiles of different marine primary producer groups visualized in multivariate space, referred to as δ^13^C_EAA_ “fingerprints”, are largely conserved across space, time, and trophic transfer (Larsen et al., 2013; McMahon et al., 2016; Elliott Smith et al., 2022). Together, δ^13^C_EAA_ and δ^15^N values of amino acids may provide the source-specific and trophic information required to trace IME-derived production from coastal to open ocean food webs.

Previous AA-CSIA applications to organisms from island ecosystems hint at this potential. In Maldivian food webs, δ^13^C_EAA_ fingerprints clearly differentiated reef-associated from pelagic planktivores (Skinner et al., 2021), and δ^15^N amino acid values have been used to resolve trophic level shifts independent of baseline nitrogen variability during onshore–offshore ontogenetic diet transitions in elasmobranchs in Kāneʻohe Bay, Oʻahu (Dale et al., 2011). Although these studies were not explicitly designed to evaluate IME processes, they collectively present AA-CSIA’s unique capacity to trace localized carbon and nitrogen inputs and quantify cross-boundary trophic connectivity in oligotrophic systems. Together, they position AA-CSIA as a strong candidate for developing a tracer for IME-derived production and for testing how nearshore nutrient subsidies shape pelagic food web structure.

In this study, we applied AA-CSIA to Hawaiian zooplankton collected along nearshore–offshore and surface–deep gradients to evaluate whether island-derived production can be traced through coastal and pelagic food webs. Specifically, we examined: (1) the extent of spatial gradients in zooplankton δ^13^C_EAA_, δ^15^N source amino acid (δ^15^N_SAA_) values, and TP with increasing distance from shore and depth, and (2) whether reef-associated zooplankton assemblages exhibit isotopic profiles distinct from offshore communities. Our results demonstrate that AA-CSIA detects island-influenced production in reef-associated zooplankton, revealing consistent isotopic differentiation from their offshore counterparts. We observed that IME-derived subsidies contribute to the trophic structure of reef-associated communities, but their influence diminishes offshore as zooplankton likely shift toward food sources characteristic of the oligotrophic open ocean. This approach provides an isotopic framework for tracing the propagation of nearshore productivity through food webs in the Hawaiian Islands, with potential for similar evaluation and application across other island and coastal systems worldwide.

## Materials & Methods

### Study sites and field sampling

**Figure 1:**
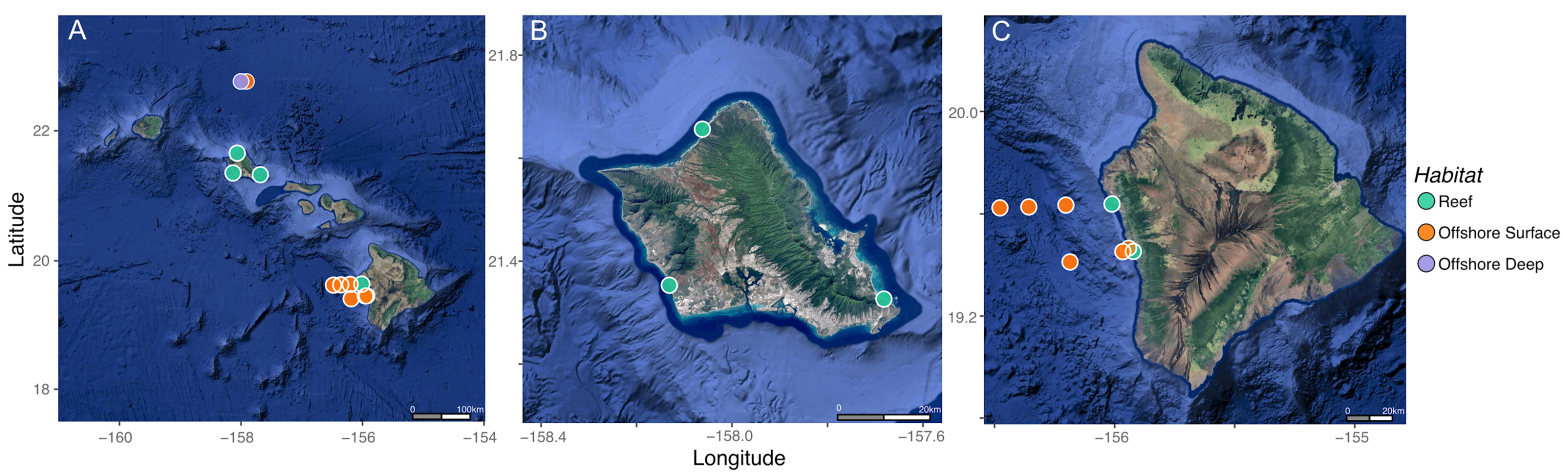
Map of sampling sites in the Hawaiian Islands. Each point represents a zooplankton tow colored by habitat type. Green points are tows collected over reefs, orange points are tows collected in offshore surface waters, and purple points are tows collected in offshore deep waters. (A) A zoomed out map of all sampling sites within the Hawaiian Archipelago. (B) A zoomed in map to show all sites from Oʻahu. (C) A zoomed in map to show all sites from Hawaiʻi Island. Satellite images copyright NASA, accessed from GoogleMaps and RgoogleMaps (Loecher & Ropkins, 2015).

This dataset comprises zooplankton collected from reef, offshore surface, and offshore deep habitats across the Hawaiian Islands over two decades (2000–2023) (Fig. 1; Table S1). Reef-associated zooplankton were defined as samples collected within one km of shore over coral reefs. Offshore surface zooplankton were defined as samples collected more than one km from shore and were further categorized by capture depth as either surface (0–200 m) or deep (>200 m). Historical offshore zooplankton AA-CSIA data were obtained from three published studies conducted at Station ALOHA, located ∼100 km north of the island of Oʻahu (Hannides et al., 2009; Hannides et al., 2013; Hannides et al., 2020). Hannides et al. (2009) used a one m diameter plankton ring net with 200 µm mesh to collect surface zooplankton, whereas Hannides et al. (2013) and Hannides et al. (2020) used a one m^2^ multiple opening/closing net and environmental sensing system (MOCNESS) with 200 µm mesh to collect zooplankton *via* oblique tows. Zooplankton samples from Hannides et al. (2013) and Hannides et al. (2020) were separated to the following size fraction bins post collection and prior to storage and analysis: 0.2–0.5 mm, 0.5–1.0 mm, 1.0–2.0 mm, and 2.0–5.0 mm. Zooplankton samples from Hannides et al. (2009) were not size fractionated prior to storage and analysis. Maximum sampling depths ranged from ∼160 m in 2000 and 2005 (Hannides et al., 2009), to 1,000 m in 2011 (Hannides et al., 2013), to 1,500 m in 2014 (Hannides et al., 2020) (Table S1). δ^13^C_EAA_ data were available only for samples collected in 2011, while δ^15^N_SAA_ and δ^15^N trophic amino acid (TAA) data were available for all Station ALOHA samples. Contemporary zooplankton were collected during 2022 and 2023 field campaigns off Oʻahu and Hawaiʻi Island using nighttime oblique tows with a one m diameter zooplankton ring net with 200 µm mesh. Reef-associated samples were collected from Electric Beach, Sunset Beach, and Kaiona Beach Park on Oʻahu in October 2022, and near Old Kona Airport Beach and near Captain Cook on Hawaiʻi Island in October 2022 and December 2023, respectively. Offshore surface samples were collected concurrently at three stations spanning an onshore–offshore gradient off the Hawaiʻi Island sampling locations using the same net. Zooplankton samples were not size fractionated prior to storage and analysis. Deep zooplankton samples were not collected during the 2022 and 2023 campaigns due to the lack of access to a MOCNESS.

### Amino acid stable isotope analysis

All zooplankton samples were processed at the Stable Isotope Biogeochemistry Laboratory at the University of Hawaiʻi at Mānoa, following established AA-CSIA protocols (Popp et al., 2007; Hannides et al., 2009). Briefly, samples were lyophilized (24–36 h), homogenized with a mortar and pestle, and hydrolyzed in trace metal grade 6 M HCl. Amino acids were isolated and purified *via* cation-exchange chromatography, esterified with a 4:1 isopropanol:acetyl chloride solution, and derivatized with 3:1 methylene chloride:trifluoroacetyl anhydride to form trifluoroacetyl amino acid esters. The resulting derivatives were purified by chloroform extraction, stored in dichloromethane-trifluoroacetic acid, and transferred to ethyl acetate for isotopic analysis.

Compound-specific isotope ratios were determined in triplicate using gas chromatography isotope ratio mass spectrometry (GC-IRMS). Amino acid δ^15^N values were measured using a Thermo Scientific Delta V Plus IRMS interfaced with a Thermo Finnigan Trace GC *via* a GC-C III combustion/reduction interface (60 m BPx5 column; 980 °C combustion, 650 °C reduction). Amino acid δ^13^C values were measured using a Thermo Scientific MAT 253 IRMS coupled to a Trace GC Ultra (30 m BPx5 column) *via* a Conflo IV interface. Analyzed amino acids for both carbon and nitrogen stable isotopes included alanine (Ala), glycine (Gly), threonine (Thr), serine (Ser), valine (Val), leucine (Leu), isoleucine (Ile), proline (Pro), methionine (Met), phenylalanine (Phe), tyrosine (Tyr), and lysine (Lys). During acid hydrolysis, asparagine (Asn) and glutamine (Gln) were converted to aspartic acid (Asp) and glutamic acid (Glu) respectively and are reported as combined Asx and Glx pools. For δ^15^N analyses, L-2-aminoadipic acid (AAA) and L-(+)-norleucine (Nor) of known isotopic composition were co-injected as internal reference compounds. δ^15^N values are expressed in δ-notation (‰) relative to atmospheric N_2_. For δ^13^C analyses, AAA, Nor, and an underivatized n-C_20_ alkane of known isotopic composition were co-injected as internal reference compounds. δ^13^C values are expressed in δ-notation (‰) relative to Vienna Pee Dee Belemnite (V-PDB). A suite of 14 amino acid reference compounds with known δ^15^N and δ^13^C values was analyzed between triplicate sample injections to apply corrections for both analyses. To correct δ^13^C values for derivatization effects, sample amino acids were calibrated against the reference suite following the approach of Silfer et al. (1991). For δ^15^N corrections, a measured *vs.* known correction curve was applied. In Hannides et al. (2009), triplicate δ^15^N AA analyses had standard deviations averaging ±0.5‰ (range: ±0.03–4.8‰). Hannides et al. (2013) reported triplicate standard deviation averages of ±0.46‰ for δ^15^N AA analyses (range: ±0.04–2.24‰) and ±0.59‰ for δ^13^C AA analyses (range: ±0.12–0.99‰). Hannides et al. (2020) reported triplicate standard deviations averaging ±0.4‰ (range: ±0.02–1.0‰) for δ^15^N AA analysis. For contemporary zooplankton samples, triplicate standard deviations averaged ±0.42‰(range: ±0.01–1.09‰) for δ^15^N AA analysis and ±0.42‰ (range: ±0.02–1.71‰) for δ^13^C AA analysis, while analytical accuracy based on amino acid reference standards averaged ±0.51‰ (range: ±0.09–1.01‰) for δ^15^N AA analysis and ±0.63‰ (range: ±0.04–1.51‰) for δ^13^C AA analysis.

### Data analyses of Hawaiian zooplankton AA-CSIA patterns

All statistical analyses were performed in R version 4.5.2 (R Core Team, 2024). Multivariate analyses were conducted using the FactoMineR (Lê, Josse & Husson, 2008) and MASS (Venables & Ripley, 2002) packages. PERMANOVA and community-level analyses were carried out using functions in the vegan (Oksanen et al., 2025) package. *Post hoc* pairwise tests and effect size calculations followed procedures implemented in rstatix (Kassambara, 2023) and FSA (Ogle, Wheeler & Dinno, 2023).

Spatial patterns in isotopic composition were evaluated using δ^13^C_EAA_, δ^15^N_SAA_, and TP estimates. δ^13^C_EAA_ values (Ile, Leu, Lys, Phe, Thr, Val) were mean-centered within each sample following Larsen et al. (2009) and Vane, Cobain & Larsen (2025). This approach facilitates comparison of endmember isotopic fingerprints among samples collected across sites, seasons, and years by removing sample-specific baseline offsets while preserving relative differences among amino acids that reflect underlying biosynthetic patterns (Elliott Smith et al., 2022). For each essential amino acid *j*, the mean-centered value was calculated as:

\begin{eqnarray*}{\delta }^{13}{C}_{j,mean-centered}={\delta }^{13}{C}_{j}-{\delta }^{13}{C}_{\mathrm{ EAA}} \end{eqnarray*}

where

\begin{eqnarray*}{\delta }^{13}{C}_{\mathrm{ EAA}}= \frac{1}{n} \sum _{i=1}^{n}{\delta }^{13}{C}_{i},\mathrm{and}~~\mathrm{n}~=~6~\mathrm{EAAs}. \end{eqnarray*}

In this formulation, *δ*^13^*C*_*j*_ is the measured δ^13^C value of essential amino acid *j* (the average value of the triplicate injections) within a given sample and *δ*^13^*C*_*j*,*mean*−*centered*_ is the corresponding mean-centered value used for statistical analysis. The term *δ*^13^*C*_EAA_ denotes the within-sample mean δ^13^C value of all essential amino acid values (again, the average value of the triplicate injections to generate each amino acid’s δ^13^C value) with *n* = 6 EAAs in this case.

For nitrogen, we used the δ^15^N values of Phe and Lys as SAAs in this study because they exhibit minimal isotopic enrichment with trophic transfer (McClelland & Montoya, 2002; McMahon & McCarthy, 2016; Shea, Drazen & Popp, 2025).

Trophic positions were estimated using a standard source-trophic amino acid framework of the form:

(1) \begin{eqnarray*}{\mathrm{TP}}_{(\mathrm{Trophic}-\mathrm{Source})}= \frac{({\delta }^{15}{N}_{Trophic}-{\delta }^{15}{N}_{Source})-\beta }{TDF} +1\end{eqnarray*}

where δ^15^N_Trophic_ is a TAA value, δ^15^N_Source_ is a SAA value, β represents the isotopic offset between these amino acids at the base of the food web, and TDF is the trophic discrimination factor describing isotopic enrichment per trophic transfer (Chikaraishi et al., 2007).

TP was calculated using two established parameterizations: TP_(Glx-Phe)_ and TP_(Ala-Phe)_. For TP_(Glx-Phe)_ we applied β = 3.4‰ and TDF = 7.6‰, values empirically derived from controlled feeding experiments across metazoan consumers (Chikaraishi et al., 2009). For TP_(Ala-Phe)_ we used β = 3.2‰ and TDF = 4.5‰, values based on laboratory calibrations that incorporate isotopic enrichment from protistan microzooplankton grazing (Décima & Landry, 2020; Shea et al., 2023). The TP_(Glx-Phe)_ formulation primarily represents metazoan trophic transfer, whereas the TP_(Ala-Phe)_ formulation additionally integrates microbial trophic steps, providing a more complete estimate of consumer trophic position in systems where protistan grazing is important (Décima et al., 2017).

Linear regressions were used to test relationships between δ^13^C_EAA_, δ^15^N_SAA_, and TP values with natural log-transformed (ln) distance from shore. Distance (in km) was derived from sample GPS coordinates and the distance to the nearest coastline calculated in Google Earth Pro (2024). Model significance was assessed using two-tailed *t*-tests and were corrected with Benjamini–Hochberg (BH) corrections for multiple comparisons. Model fit was evaluated using R^2^.

To compare isotopic and trophic metrics among grouped reef, offshore surface, and offshore deep assemblages, data were tested for normality using a Shapiro–Wilk test and for homogeneity of variance using a Levene’s test. When assumptions for normality were met, one-way ANOVA tests were used to compare the three groups and were followed by Tukey’s *post hoc* tests if ANOVA results were significant (*p* < 0.05). When assumptions for normality were not met, nonparametric Kruskal–Wallis tests were used to compare the three groups and followed by BH-corrected Dunn’s *post hoc* comparisons if Kruskal–Wallis results were significant (*p* < 0.05).

Multivariate differences across δ^13^C_EAA_, δ^15^N_SAA_, and TP values between the three groups were assessed using permutational multivariate analysis of variance (PERMANOVA) based on Euclidean distances. Homogeneity of dispersion was assessed before interpreting PERMANOVA results. When significant group effects were detected (*p* < 0.05) and dispersion effects were non-significant (*p* > 0.05), pairwise PERMANOVA tests were used to identify specific group contrasts. Multivariate separation was visualized using linear discriminant analysis (LDA), and variable loadings were examined to identify isotopic metrics contributing most to group separation. Leave-one-out cross-validation (LOOCV) was used to assess classification accuracy, though not as a formal test of significance.

### Maldivian consumer δ^13^C_EAA_ patterns

To evaluate whether nearshore–offshore isotopic differences occur in consumers in other oligotrophic island systems, the same univariate framework was applied to published δ^13^C_EAA_ data for Maldivian planktivores (Skinner et al., 2021). Because Ile was not reported in the original study, δ^13^C values were mean-centered across five EAAs (Thr, Val, Leu, Phe, Lys). Planktivores were grouped by presumed diet as in the original study: fusiliers and soldierfish as reef planktivores, and deep-living squid and mackerel scad as pelagic planktivores. Each variable was tested for normality and homogeneity of variance using Shapiro–Wilk and Levene’s tests. Welch’s *t*-tests were used to compare reef *vs.* pelagic planktivores when assumptions for normality were met, and Mann–Whitney U tests were used otherwise. All *p*-values were BH-corrected for multiple comparisons.

### Primary producer community drivers of zooplankton δ^13^C_EAA_ patterns

To infer basal carbon sources supporting zooplankton, and to investigate how δ^13^C_EAA_ gradients vary with distance from shore, a supervised classification LDA approach was performed using published δ^13^C_EAA_ data from cultured phytoplankton (Stahl, Rynearson & McMahon, 2023) representing diatoms, dinoflagellates, prasinophytes, and raphidophytes as training endmembers. Training data were mean-centered across six EAAs (Ile, Leu, Lys, Phe, Thr, Val). Hawaiian zooplankton samples were then classified relative to these producer groups using their mean-centered δ^13^C_EAA_ values. Despite its exclusion in Stahl, Rynearson & McMahon (2023), we retained Lys in our LDA because it improved source discrimination between reef and offshore zooplankton. The Maldivian dataset was excluded for LDA analysis because Ile was not reported. When Ile was omitted from the training dataset to enable classification of Maldivian consumers, diatom and prasinophyte groups overlapped significantly, thereby reducing classification power and precluding meaningful comparisons.

## Results

### δ^13^C_EAA_ values of Hawaiian zooplankton nearshore to offshore and with depth

**Figure 2:**
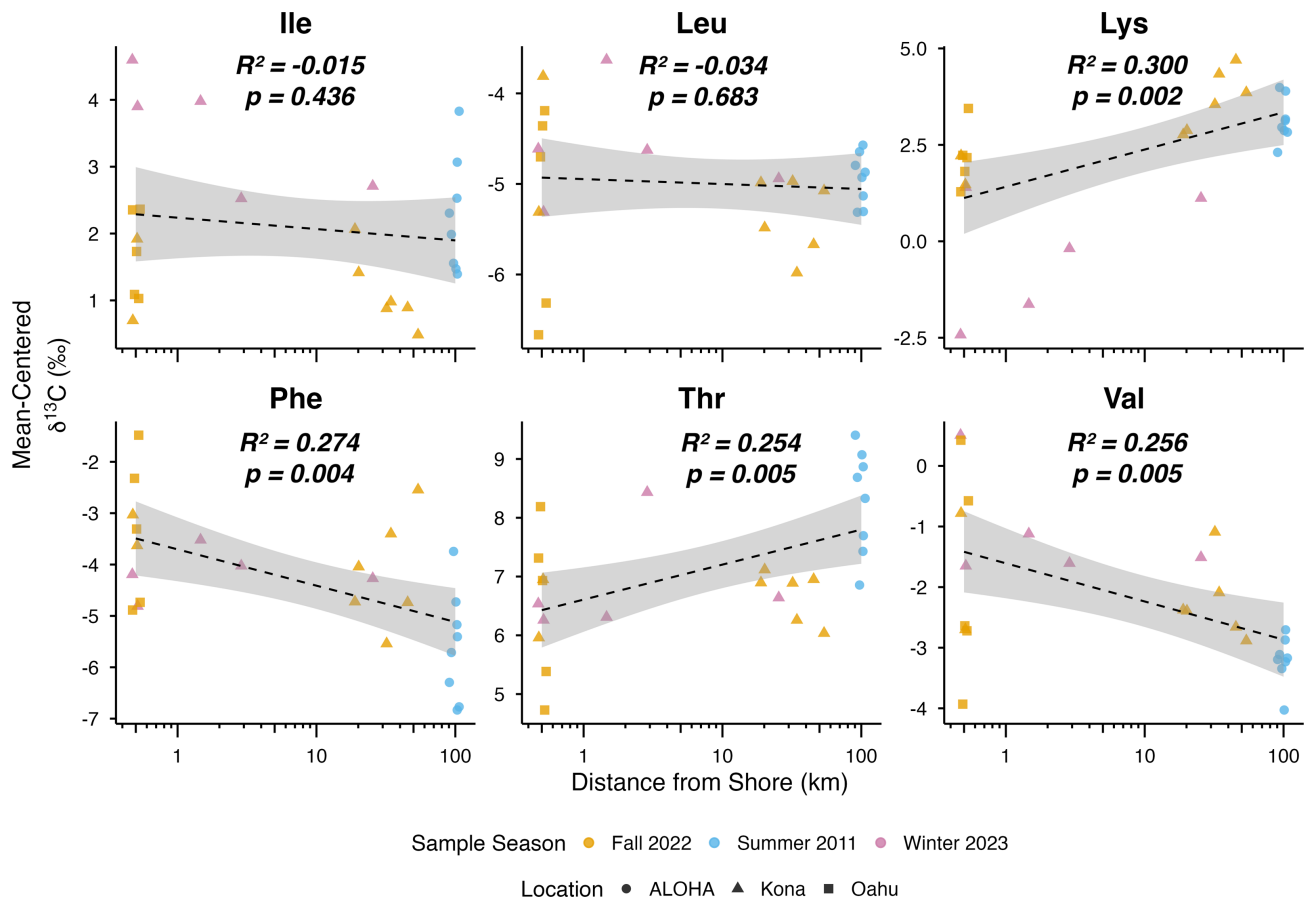
Distance from shore (km) as a linear predictor of mean-centered δ^13^C_EAA_ values (Ile, Leu, Lys, Phe, Thr, and Val) of Hawaiian zooplankton. Tick marks on the *x*-axis show natural log (ln) transformed distance from shore, while number labels denote true values in km. Adjusted *R*^2^ values and adjusted *p*-values are reported for each amino acid. Significant *p*-values are in bold. Colors indicate season of collection, and symbols denote sampling locations.

Linear regression analyses revealed significant relationships between δ^13^C values and natural log-transformed distance from shore for four of the six EAAs measured in Hawaiian zooplankton (Fig. 2; Table S2). δ^13^C values of Phe and Val declined significantly with increasing offshore distance (*p* = 0.004 and *p* = 0.005 respectively), whereas those of Thr and Lys showed significant increases (*p* = 0.005 and *p* = 0.002 respectively). In contrast, Ile and Leu remained unchanged with distance from shore (*p* = 0.436 and *p* = 0.683 respectively).

**Figure 3:**
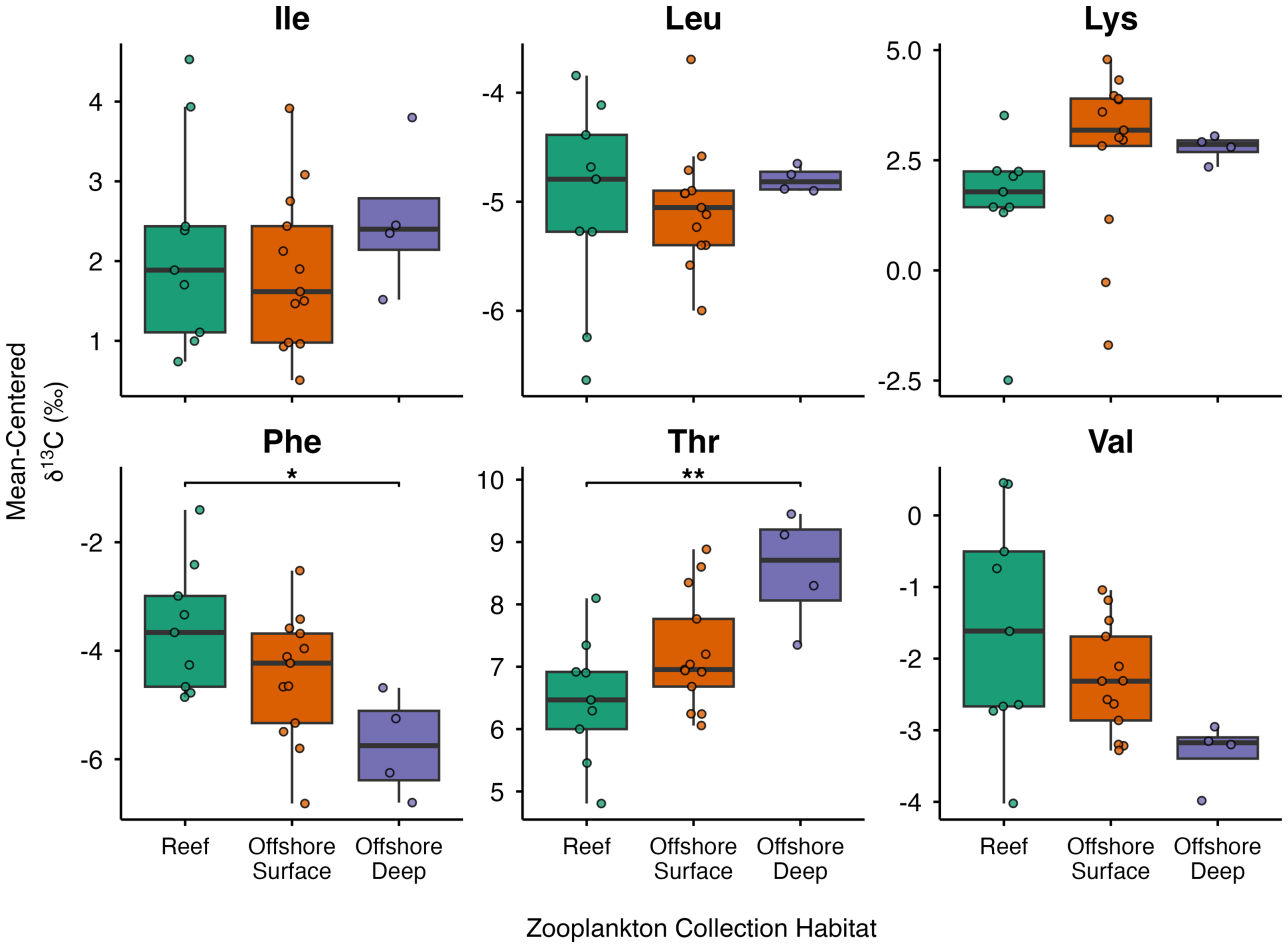
Boxplots of mean-centered δ^13^C_EAA_ values (Ile, Leu, Lys, Phe, Thr, and Val) from Hawaiian reef (*n* = 9), offshore surface (*n* = 13), and offshore deep (*n* = 4) zooplankton. Colors indicate different zooplankton collection habitats. Horizontal bars represent significant pairwise comparisons, with * denoting *p* < 0.05 and ** denoting *p* < 0.01.

To further examine spatial and depth-related variation in δ^13^C_EAA_ values, Hawaiian zooplankton samples were compared across three groups: reef, offshore surface, and offshore deep habitats (Fig. 3; Table S3). Significant differences among habitats were detected for Phe (*p* = 0.015) and Thr (*p* = 0.005), while Val (*p* = 0.052) and Lys (*p* = 0.050) exhibited marginally non-significant differences among habitats. In contrast, Ile (*p* = 0.537) and Leu (*p* = 0.814) did not vary significantly among habitats. *Post hoc* comparisons revealed that reef zooplankton had significantly higher Phe values than offshore deep zooplankton (*p* = 0.012), whereas Thr values were significantly lower in reef compared to offshore deep zooplankton (*p* = 0.004).

### δ^13^C_EAA_ values of Maldivian reef and pelagic planktivores

To evaluate whether the same nearshore–offshore patterns in plankton δ^13^C_EAA_ variation also occur in consumers from other oligotrophic ocean regions, Maldivian planktivores were grouped by presumed diet (reef *vs.* pelagic plankton) and compared (Fig. S1; Table S4). Phe and Leu values were significantly higher in reef consumers (*p* < 0.001 and *p* = 0.002), whereas Thr and Lys values were significantly lower in reef consumers (*p* < 0.001 and *p* = 0.004). Val showed no significant difference between consumer groups (*p* = 0.374).

### Linear discriminant analysis: δ^13^C_EAA_ values of Hawaiian zooplankton

An LDA of δ^13^C_EAA_ values in Hawaiian zooplankton revealed separation among reef and offshore deep assemblages along the first discriminant axis (LD1), which accounted for 92.8% of the total between-group variance (Fig. S2). The second axis (LD2) explained the remaining 7.2% of variance. PERMANOVA results indicated significant isotopic differentiation among groups (*p* = 0.007; Table S5) and the test for dispersion homogeneity was not significant (*p* = 0.253). Pairwise PERMANOVA showed that the significant effect was driven primarily by differences between reef and offshore deep zooplankton (*p* = 0.003). Loadings on LD1 were primarily influenced by δ^13^C values of Leu and Phe, with moderate contributions from δ^13^C values of Thr and Val. LOOCV yielded a classification accuracy of 42.3%, indicating limited predictive power.

### Linear discriminant analysis: δ^13^C_EAA_ values of Hawaiian zooplankton compared to cultured primary producers

**Figure 4:**
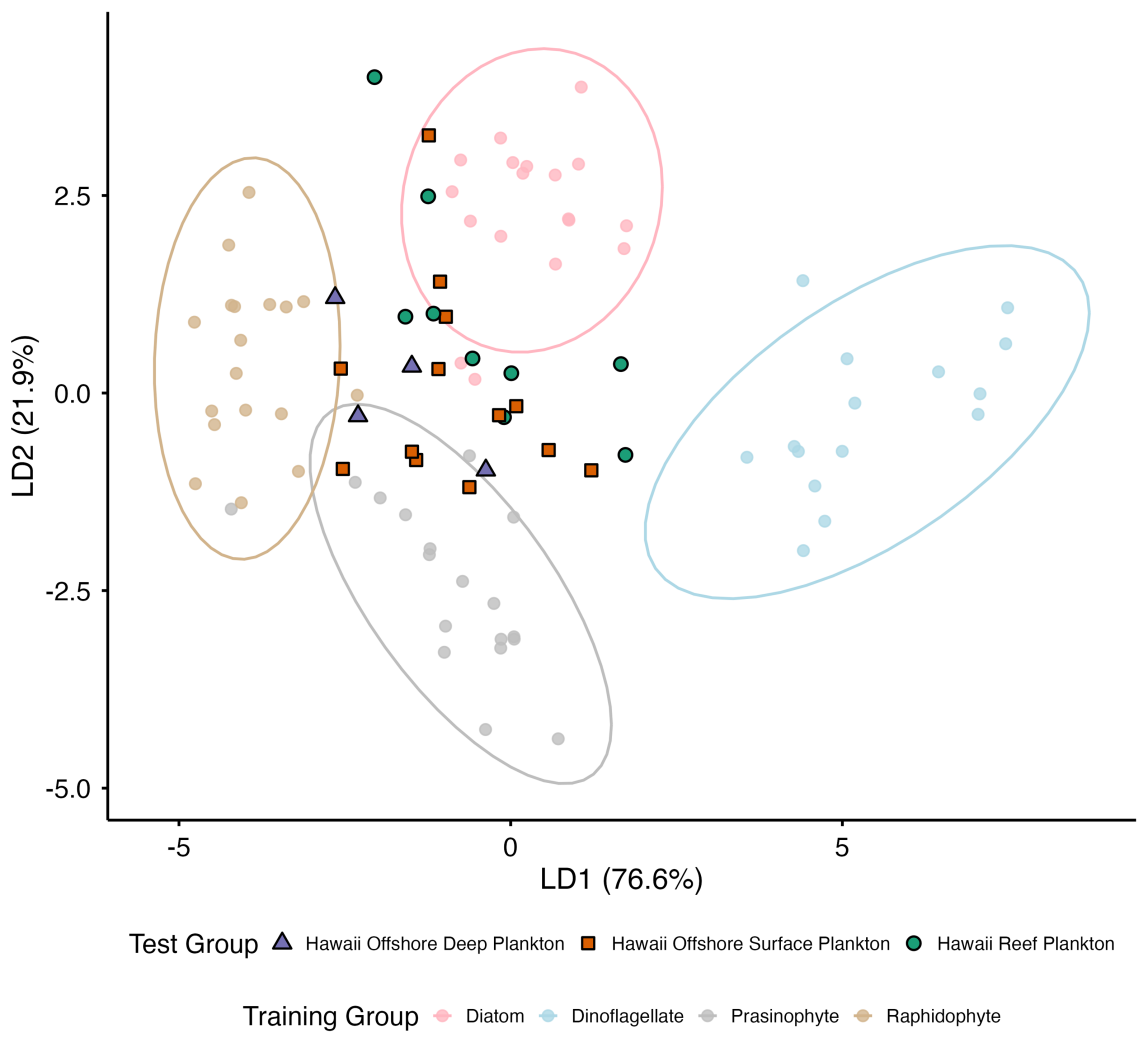
LDA based on mean-centered δ^13^C_EAA_ values (Ile, Leu, Lys, Phe, Thr, and Val) from four cultured marine primary producer groups (Stahl, Rynearson & McMahon, 2023) used as training data. Hawaiian zooplankton samples were projected into the same discriminant space as test data to assess isotopic similarity to producer end-members. Black-outlined colored shapes indicate different zooplankton collection habitats, while faded colored circles represent the different marine primary producer groups. Faded colored ellipses represent 95% confidence intervals around producer groups.

Using LDA on the δ^13^C_EAA_ values of Thr, Ile, Val, Phe, and Leu from cultured diatoms, dinoflagellates, prasinophytes, and raphidophytes, Stahl, Rynearson & McMahon (2023) showed that these amino- acid δ^13^C patterns effectively distinguish the four primary- producer groups. Here, we incorporate δ^13^C Lys values, which further enhance group separation (Fig. 4). PERMANOVA results indicated significant isotopic differentiation among groups (*p* = 0.001; Table S6) and the test for dispersion homogeneity was not significant (*p* = 0.887). Pairwise PERMANOVA showed that all primary producer groups were significantly different from one another (*p* = 0.001). LD1 and LD2 explained 76.6% and 21.9% of the between-group variance with δ^13^C values of Phe, Leu, and Val contributing most strongly to group separation along LD1, and δ^13^C values of Ile, Thr, and Lys contributing most strongly to LD2. LOOCV of the training dataset yielded an overall classification accuracy of 95.65%, similar to results presented in Stahl, Rynearson & McMahon (2023).

When Hawaiian zooplankton samples were projected into the LDA space (Fig. 4), most reef zooplankton samples (seven of nine) were classified as diatom (posterior probabilities = 0.83–1.00), while two of nine samples were classified as prasinophyte (0.65–0.67). Among the 13 offshore surface zooplankton samples, most samples (seven of thirteen)) were classified as prasinophyte (0.66–1.00), five as diatom (0.57–1.00), and one as raphidophyte (0.91). For the four offshore deep plankton samples, two were identified as prasinophyte (0.53 and 0.99), one as raphidophyte (0.82), and one as diatom (0.65).

### δ^15^N_SAA_ values of Hawaiian zooplankton nearshore to offshore and with depth

**Figure 5:**
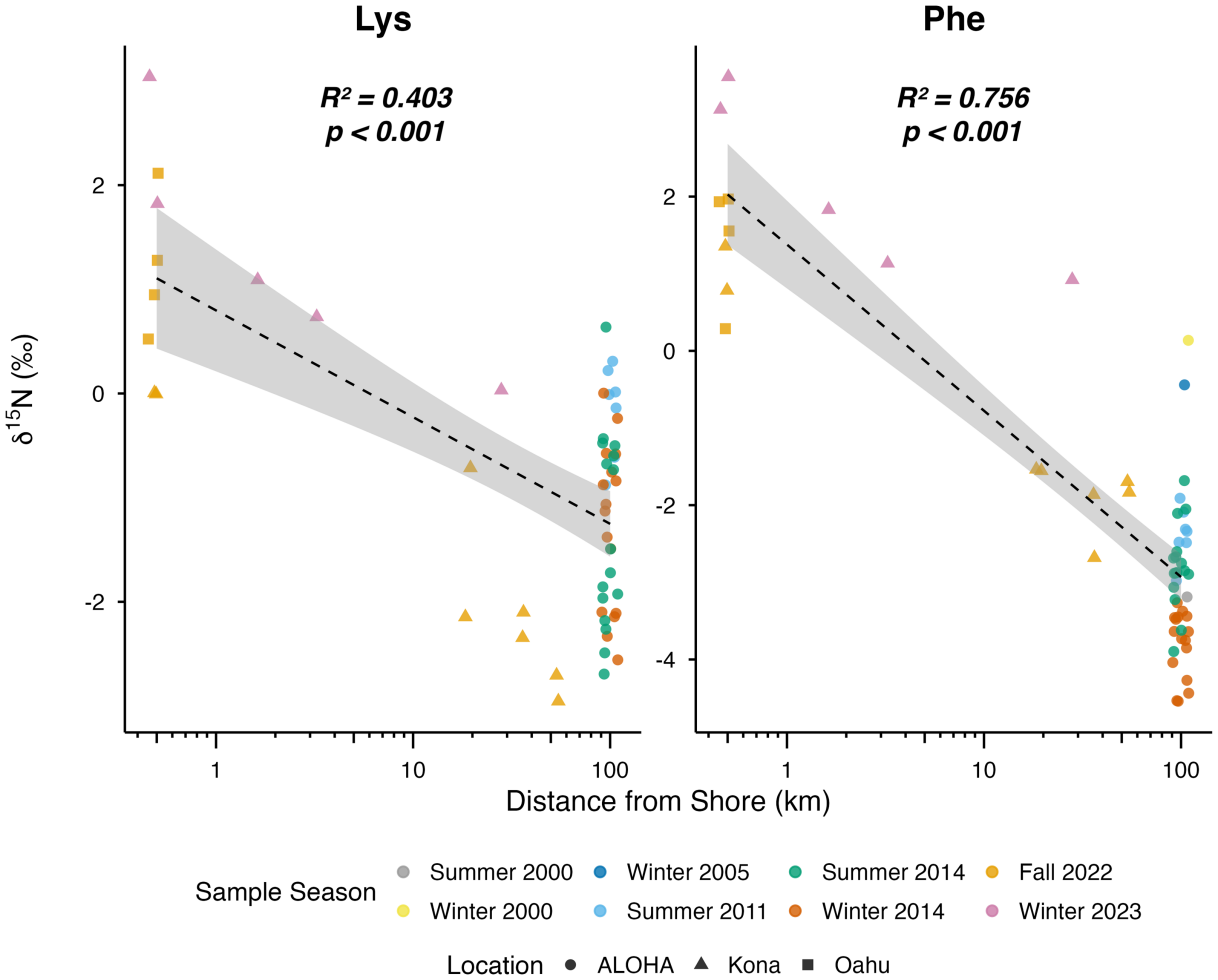
Distance from shore (km) as a linear predictor of δ^15^N_SAA_ values (Lys, Phe) of Hawaiian zooplankton. Offshore deep samples were removed due to similarity to reef values. Tick marks on *x*-axis show natural log (ln) transformed distance from shore; number labels denote true values in km. Adjusted *R*^2^ values and adjusted *p*-values are reported for each amino acid. Significant *p*-values are in bold. Colors indicate season of collection, and symbols denote sampling locations.

Linear regression analyses revealed significant relationships between distance from shore and both δ^15^N Phe and δ^15^N Lys values in Hawaiian zooplankton collected in surface waters (Fig. 5; Table S7). Phe and Lys values both declined significantly with increasing distance from shore (*p* < 0.001 for both).

**Figure 6:**
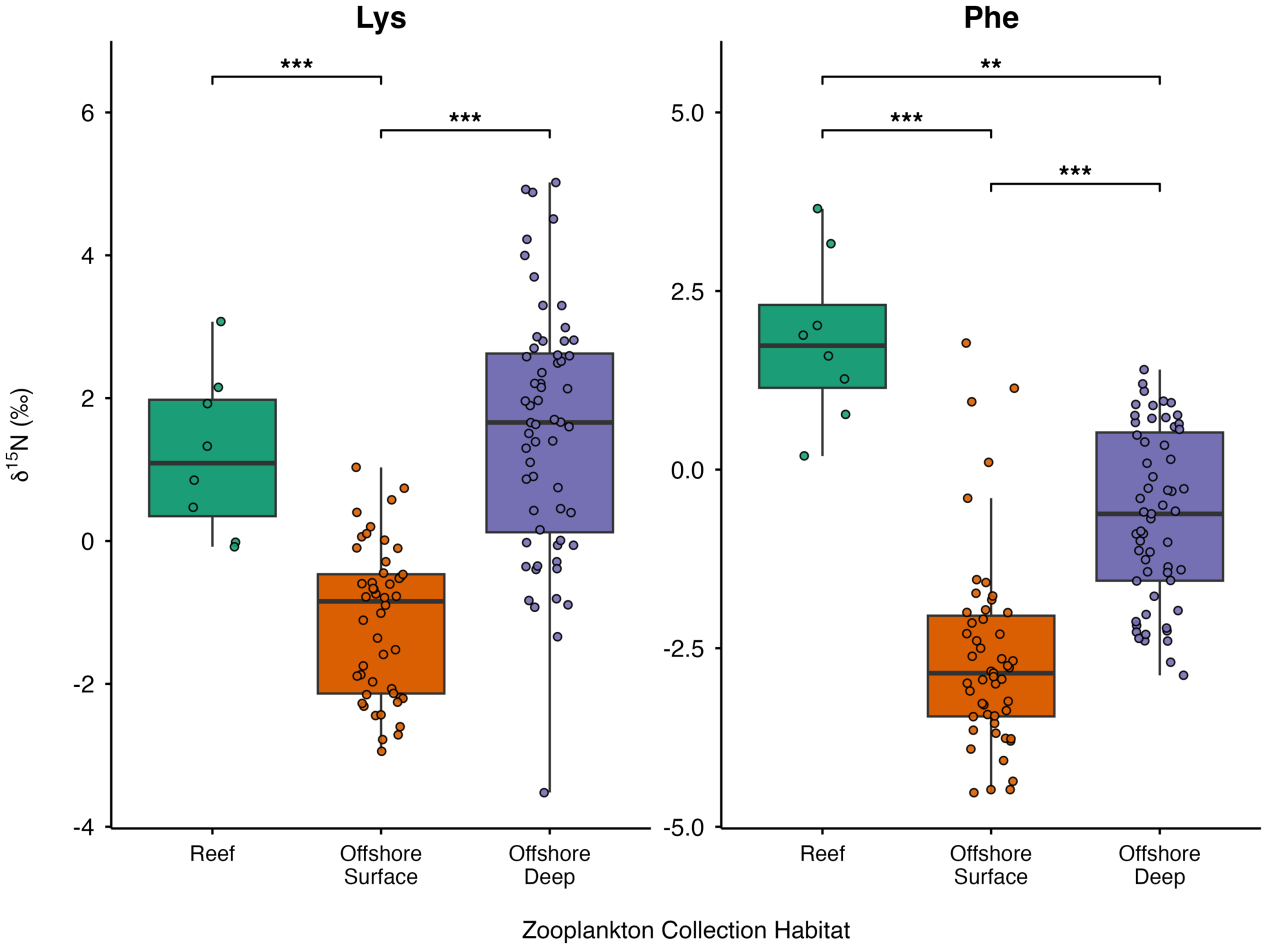
Boxplots of δ^15^N_SAA_ values (Lys and Phe) from Hawaiian reef (*n* = 9), offshore surface (*n* = 51), and offshore deep (*n* = 60) zooplankton. Horizontal bars represent significant pairwise comparisons, with ** denoting *p* < 0.01 and *** denoting *p* < 0.001.

To assess spatial and depth-related variation in δ^15^N_SAA_ values, Hawaiian zooplankton samples were further compared across reef, offshore surface, and offshore deep collection habitats (Fig. 6; Table S8). Significant differences among collection habitats were detected for both Phe and Lys (*p* < 0.001 for both). Pairwise comparisons revealed that offshore surface zooplankton exhibited significantly lower Phe values than reef zooplankton (*p* <0.001) and offshore deep zooplankton (*p* < 0.001), and Lys values were similarly significantly lower in offshore surface zooplankton relative to reef zooplankton (*p* < 0.001) and to offshore deep zooplankton (*p* < 0.001). Further, reef zooplankton had significantly higher Phe values than offshore deep zooplankton (*p* = 0.006).

### Linear discriminant analysis: δ^13^C_EAA_ and δ^15^N_SAA_ values of Hawaiian zooplankton

**Figure 7:**
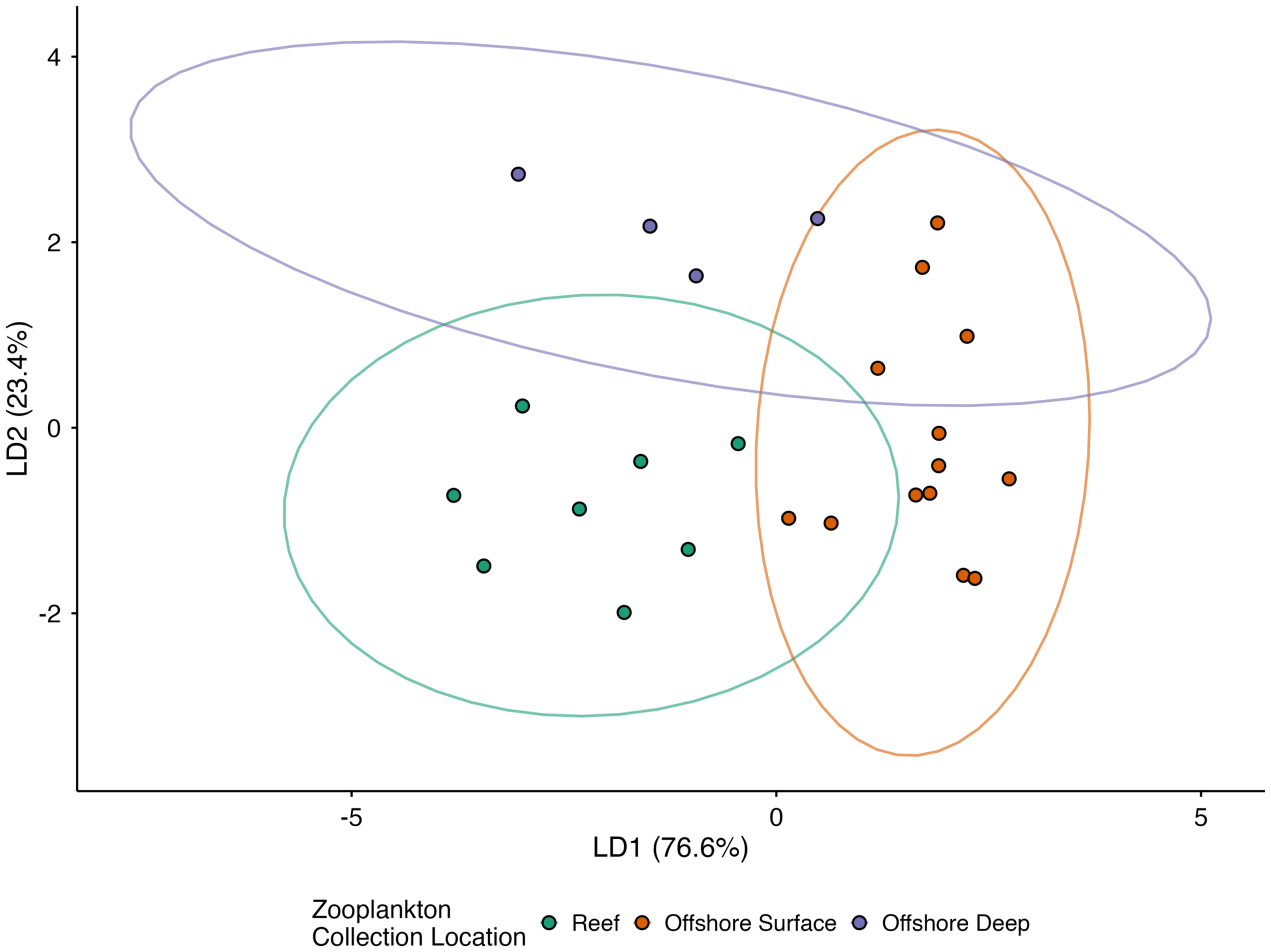
LDA of Hawaiian zooplankton mean-centered δ^13^C_EAA_ values (Thr, Val, Leu, Ile, Phe, Lys) and δ^15^N_SAA_ values (Lys, Phe) between reef, offshore surface, and offshore deep habitats. Pairwise PERMANOVA revealed significant differences between all groups. Colored ellipses represent 95% confidence intervals around habitat groups.

The LDA integrating δ^13^C_EAA_ and δ^15^N_SAA_ values revealed clear separation among reef, offshore surface, and offshore deep zooplankton assemblages (Fig. 7). LD1 accounted for 76.6% of the total between group variance, while LD2 explained the remaining 23.4%. PERMANOVA results confirmed significant isotopic differentiation among groups (*p* = 0.001), and the test for homogeneity of dispersion was not significant (*p* = 0.485) (Table S9). Pairwise PERMANOVA identified significant differences among all group pairs: reef *vs.* offshore surface (*p* = 0.001), offshore surface *vs.* offshore deep (*p* = 0.002), and reef *vs.* offshore deep (*p* = 0.003). Loadings on LD1 were most strongly influenced by δ^15^N values of Phe and Lys, and δ^13^C values of Thr and Lys. Loadings on LD2 were most strongly influenced by δ^15^N values of Lys and δ^13^C values of Leu Phe, and Val. LOOCV yielded a classification accuracy of 80%, correctly identifying most samples within their respective habitat groups.

### Trophic position estimates of Hawaiian zooplankton nearshore to offshore and with depth

**Figure 8:**
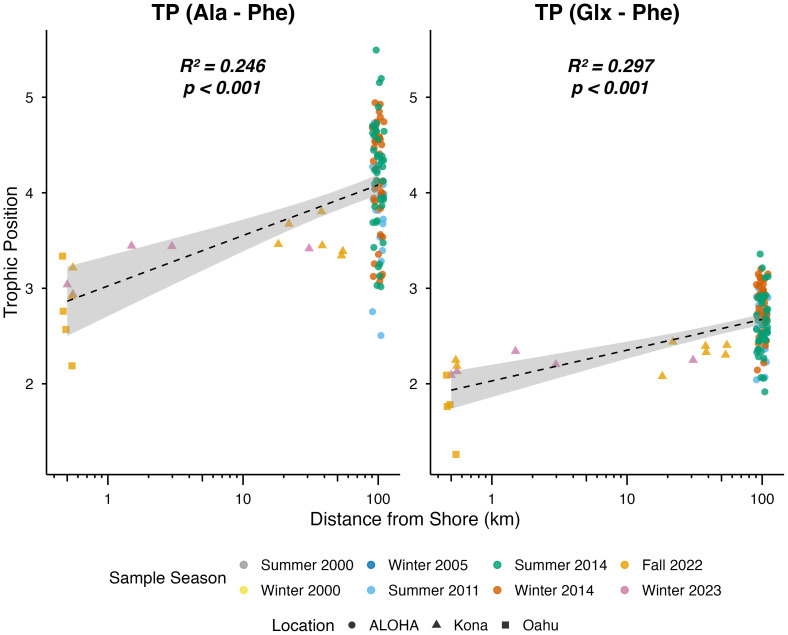
Distance from shore (km) as a linear predictor of zooplankton trophic position (TP_(Ala-Phe)_ and TP_(Glx-Phe)_) of Hawaiian zooplankton. Tick marks on *x*-axis show natural log (ln) transformed distance from shore; number labels denote true values in km. Adjusted *R*^2^ values and adjusted *p*-values are reported for each amino acid. Significant *p*-values are in bold. Colors indicate season of collection, and symbols denote sampling locations.

Linear regression analyses revealed significant positive relationships between Hawaiian zooplankton TP and natural log-transformed distance from shore for both TP_(Ala-Phe)_ and TP_(Glx-Phe)_ estimates (*p* < 0.001 in both cases) (Fig. 8; Table S10).

**Figure 9:**
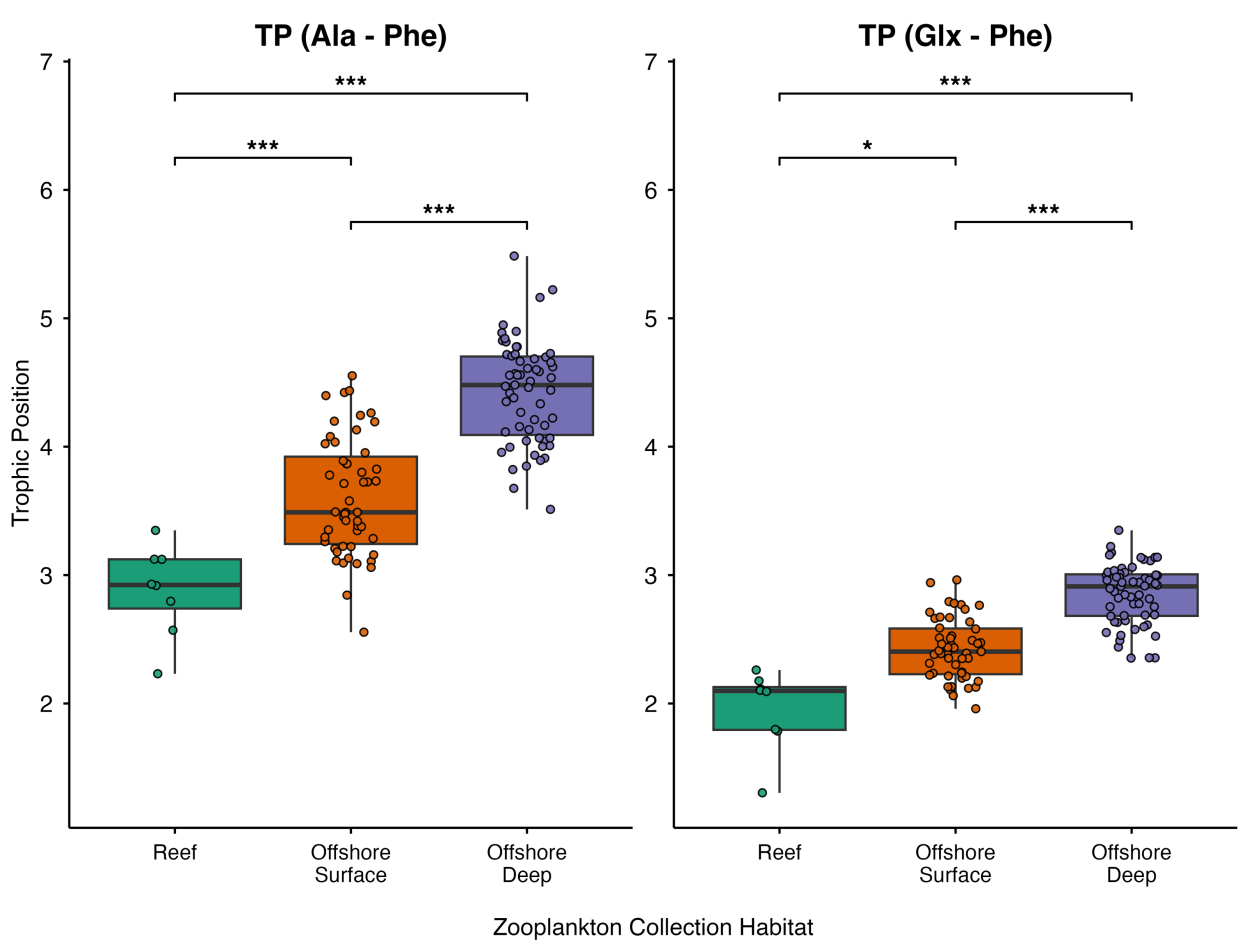
Boxplots of zooplankton trophic position (TP_(Ala-Phe)_ and TP_(Glx-Phe)_) from Hawaiian reef (*n* = 8), offshore surface (*n* = 51), and offshore deep (*n* = 59) zooplankton. Horizontal bars represent significant pairwise comparisons, with * denoting *p* < 0.05, ** denoting *p* < 0.01, and *** denoting *p* < 0.001.

Significant spatial and depth-related variation were also observed in zooplankton TP estimates (Fig. 9; Table S11). Significant differences among collection habitats were detected for both TP_(Ala-Phe)_ and TP_(Glx-Phe)_ (*p* < 0.001 for both). Pairwise comparisons revealed that reef zooplankton exhibited significantly lower TP_(Glx-Phe)_ estimates compared to offshore surface zooplankton (*p* = 0.016) and offshore deep zooplankton (*p* < 0.001), with TP_(Ala-Phe)_ mirroring these results of significantly lower estimates in reef than offshore surface (*p* < 0.001) and offshore deep (*p* < 0.001) zooplankton. Further, offshore surface zooplankton had significantly lower TP_(Glx-Phe)_ and TP_(Ala-Phe)_ estimates than offshore deep zooplankton (*p* < 0.001 in both cases).

### Linear discriminant analysis: δ^13^C_EAA_ values, δ^15^N_SAA_ values, and TP estimates of Hawaiian zooplankton

An LDA integrating δ^13^C_EAA_ values, δ^15^N_SAA_ values, and TP estimates revealed pronounced isotopic separation among reef, offshore surface, and offshore deep zooplankton assemblages (Fig. S3, Table S12). LD1 accounted for 57.3% of the total between-group variance, while LD2 explained the remaining 42.7%. PERMANOVA confirmed significant differences between groups (*p* = 0.001), and the test for homogeneity of dispersion was not significant (*p* = 0.456). Pairwise PERMANOVA comparisons identified significant differences between all group pairs: reef *versus* offshore surface (*p* = 0.001), reef *versus* offshore deep (*p* = 0.006), and offshore surface *versus* offshore deep (*p* = 0.001). Loadings on LD1 were most strongly influenced by TP_(Ala-Phe)_, TP_(Glx-Phe)_, and δ^13^C values of Val and Leu. Loadings on LD2 were most strongly influenced by TP_(Ala-Phe)_, TP_(Glx-Phe)_, δ^13^C values of Thr, and δ^15^N values of Lys. LOOCV yielded a classification accuracy of 80%, correctly identifying most samples within their respective habitat groups, but not markedly improving classification accuracy from the LDA with just δ^13^C_EAA_ and δ^15^N_SAA_ values.

## Discussion

This study applied AA-CSIA to Hawaiian zooplankton to evaluate whether island-derived production leaves detectable isotopic compositions traceable into consumers across nearshore–offshore gradients. To provide an ecologically grounded framework for interpreting these patterns, we defined reef-associated and offshore zooplankton based on spatial structure in δ^15^N_SAA_ values across distance-from-shore and tow-depth gradients within the study region. Zooplankton collected in surface waters over coral reefs within one km of shore consistently exhibited elevated δ^15^N_SAA_ values, whereas individuals collected more than 1 km offshore in surface waters (including intermediate distances of 1.5 and three km from shore) displayed consistently lower δ^15^N_SAA_ values similar to those collected at >20 km from shore. In the steep-slope island settings examined here, where bottom depths reach ∼1,000 m within a few kilometers of shore, these observations indicate a rapid attenuation of island-associated isotopic signatures with increasing distance from shore. Accordingly, samples collected <1 km from shore in this study were classified as reef-associated, while those collected >1 km from shore were classified as offshore. However, the magnitude and offshore extent of the isotopic compositions characteristic of island production may persist farther than 1.5–3 km in different systems depending on factors such as geomorphic type (atoll *vs.* island), bathymetric slope, reef area, and local human impacts (Gove et al., 2016). Offshore surface and offshore deep assemblages were further distinguished based on depth-related shifts in nitrogen cycling, with zooplankton collected below 200 m categorized as offshore deep. This depth threshold is consistent with prior work at Station ALOHA showing elevated zooplankton δ^15^N_SAA_ values below ∼150 m due to increased reliance on microbially reworked particulate organic matter (Hannides et al., 2013; Hannides et al., 2020). Together, these distance- and depth-based thresholds provide a biologically informed framework for interpreting isotopic gradients associated with the IME at our study sites.

Multivariate analyses integrating δ^13^C_EAA_ and δ^15^N_SAA_ values revealed that reef-associated zooplankton possess distinct isotopic profiles relative to offshore surface and offshore deep assemblages. This separation indicates that IME-enhanced productivity generates a measurable isotopic imprint that is retained in nearshore consumers. Because δ^13^C_EAA_ and δ^15^N_SAA_ values undergo minimal fractionation during trophic transfer, these isotopic values provide conservative tracers of island-derived production into zooplankton and, potentially, into planktivores and higher trophic level consumers that exploit island-enhanced food subsidies. Collectively, these results demonstrate the utility of AA-CSIA for linking IME-driven nutrient inputs to food web structure and establish a foundation for assessing how δ^13^C_EAA_, δ^15^N_SAA_, and TP each capture distinct components of the nearshore–offshore productivity gradient.

The nearshore–offshore gradients in zooplankton δ^13^C_EAA_ values likely reflect shifts in primary producer community composition driven by IME-enhanced nutrient supply. Nutrient-rich coastal Hawaiian waters are typically dominated by larger-celled phytoplankton such as diatoms, dinoflagellates, and cryptophytes, along with smaller cyanobacteria like *Synechococcus* (Tucker et al., 2025). In contrast, offshore oligotrophic waters are dominated by smaller cyanobacteria and pico- to nano-phytoplankton, including *Prochlorococcus*, *Trichodesmium*, picoprasinophytes, prymnesiophytes, and pelagophytes (Carpenter et al., 1997; Worden, Nolan & Palenik, 2004; Limardo et al., 2017). These taxonomic transitions likely underlie the observed zooplankton δ^13^C_EAA_ patterns due to distinct biochemical pathways for amino acid synthesis in different phytoplankton taxa. For example, cyanobacteria and eukaryotic phytoplankton synthesize EAAs *via* distinct metabolic pathways, resulting in taxonomically diagnostic δ^13^C_EAA_ fingerprints (Larsen et al., 2009; Stahl, Rynearson & McMahon, 2023; Vane, Cobain & Larsen, 2025). Accordingly, the increased availability of cyanobacteria offshore and larger eukaryotic phytoplankton nearshore likely drives a shift in zooplankton diet along this gradient, integrating the isotopic fingerprints of their dominant prey. Together, these results suggest that both phytoplankton community composition and underlying biosynthetic differences contribute to the isotopic structuring of zooplankton communities from reef-associated to offshore habitats.

Our primary producer LDA supports this interpretation, revealing nearshore–offshore structuring, with reef-associated zooplankton aligning most closely with diatom end-members and offshore assemblages aligning most closely with prasinophyte sources. However, current primary producer δ^13^C_EAA_ reference libraries remain incomplete, lacking several ecologically important taxa in the North Pacific Subtropical Gyre. These include cyanobacteria (*e.g.*, *Prochlorococcus*, *Synechococcus*, *Trichodesmium*), diatoms hosting N_2_-fixing symbionts (*e.g.*, *Hemiaulus, Rhizosolenia, Epithemia*), and other aforementioned phytoplankton groups that likely contribute to zooplankton diets along IME gradients in the Hawaiian Islands (Villareal, 1991; Schvarcz et al., 2022). In addition, selective feeding by zooplankton may further influence δ^13^C_EAA_ patterns, as phytoplankton abundance along the nearshore–offshore gradient may not necessarily reflect dietary preference (Teegarden, Campbell & Durbin, 2001). Expanding δ^13^C_EAA_ libraries to include regionally relevant taxa would improve source assignment, help resolve zooplankton feeding selectivity, and clarify which phytoplankton transitions most strongly drive observed δ^13^C_EAA_ gradients. Although several individual EAAs exhibited strong nearshore–offshore trends, multivariate δ^13^C_EAA_ profiles alone did not fully discriminate Hawaiian reef, offshore surface, and offshore deep zooplankton assemblages, indicating that δ^13^C_EAA_ must be paired with complementary tracers to confidently resolve IME influences.

To evaluate whether these patterns are generalizable across island systems, we compared Hawaiian zooplankton δ^13^C_EAA_ values with published δ^13^C_EAA_ data from Maldivian planktivores. Nearshore–offshore patterns in δ^13^C values of Phe, Thr, and Lys in Maldivian planktivores closely resemble those observed in Hawaiian zooplankton, suggesting that nutrient-driven shifts in phytoplankton community composition can structure consumer δ^13^C_EAA_ profiles across island ecosystems. In contrast, Leu and Val exhibited less consistent trends between the two systems. These differences may reflect regional variation in dominant phytoplankton taxa along nearshore–offshore gradients or may result from the Maldivian dataset representing higher trophic level consumers (planktivores) rather than zooplankton, whose more diverse diets may partially obscure purely primary producer isotopic patterns. This comparison highlights the need for further studies that jointly resolve phytoplankton, zooplankton, and higher trophic level consumer community composition, diet studies, and AA-CSIA studies that track δ^13^C_EAA_ transfer through trophic levels across different island systems. Despite these inconsistencies, the broadly similar patterns observed for most EAAs indicate that δ^13^C_EAA_ values provide a partial tracer of nearshore–offshore food sources in both the Maldives and the Hawaiian Islands, lending further support to the hypothesis that IME-driven phytoplankton community shifts drive nearshore–offshore consumer δ^13^C_EAA_ gradients.

Complementary δ^15^N_SAA_ patterns revealed pronounced differences in nitrogen source utilization between reef-associated and offshore zooplankton assemblages. Offshore surface zooplankton exhibited low δ^15^N_SAA_ values, consistent with nitrogen supplied by N_2_ fixation and tight microbial regeneration loops in offshore oligotrophic waters that depress δ^15^N values (Dore et al., 2002; McClelland, Holl & Montoya, 2003). In contrast, reef-associated zooplankton showed elevated δ^15^N_SAA_ values, reflecting the possible influence of multiple ^15^N-enriched nitrogen sources including partially denitrified soil nitrate delivered *via* surface runoff (Cox et al., 2013), sewage effluent (Aravena, Evans & Cherry, 1993; Dailer et al., 2012; Aguiar et al., 2023), submarine groundwater discharge (Fackrell et al., 2016; Bishop et al., 2017), and episodic inputs of upwelled deep water (Casciotti et al., 2008). Offshore deep zooplankton also exhibited elevated δ^15^N_SAA_ values, consistent with reliance on microbially reworked sinking organic matter at depth (Hannides et al., 2013; Romero-Romero et al., 2021). Because reef-associated and offshore deep assemblages share similarly high δ^15^N_SAA_ values, δ^15^N_SAA_ values alone cannot distinguish island-derived nitrogen inputs from deep recycled sources. However, combining δ^13^C_EAA_ and δ^15^N_SAA_ values in multivariate space substantially improves discrimination among reef-associated, offshore surface, and offshore deep zooplankton assemblages.

Differences in reef-associated and offshore zooplankton TP estimates (TP_(Glx-Phe)_ and TP_(Ala-Phe)_ revealed systematic shifts in food web structure across the IME gradient. Offshore zooplankton exhibited higher TP values, consistent with the longer food webs characteristic of oligotrophic environments where small primary producers support additional protistan and microzooplankton trophic steps before reaching mesozooplankton (Lalli & Parsons, 1997). These microbial grazing pathways are well documented in the North Pacific Subtropical Gyre (Landry et al., 2008) and have been shown to influence zooplankton amino acid composition in mesoscale eddy systems (Décima & Landry, 2020). In contrast, IME-enhanced coastal productivity favors larger primary producers and more direct trophic pathways to mesozooplankton (Comfort et al., 2024), resulting in lower TPs for reef-associated zooplankton. TP_(Ala-Phe)_ values were consistently ∼1 trophic level higher than TP_(Glx-Phe)_ values, likely reflecting the greater sensitivity of Ala-based estimates to additional microbial processing within the food web (Shea et al., 2023). Together, these TP patterns complement δ^13^C_EAA_ and δ^15^N_SAA_ results, indicating that IME-driven productivity influences not only basal carbon and nitrogen resources but also the trophic architecture of pelagic consumer communities.

Although clear IME-related gradients emerged, isotopic patterns must be interpreted in the context of dynamic oceanographic variability. Mesoscale features such as cyclonic eddies can temporarily alter δ^13^C_EAA_ and δ^15^N_SAA_ baselines through the injection of deep, nutrient-rich waters that alter primary producer community compositions in offshore surface waters (Calil et al., 2008; Landry et al., 2008; Rii et al., 2008). Notably, one of our sampling campaigns near Hawaiʻi Island (winter 2023) occurred shortly after a cyclonic eddy event, during which offshore zooplankton exhibited elevated δ^15^N_SAA_ values and lower δ^13^C Lys values compared to offshore zooplankton sampled in a nearby region in fall 2022. These shifts likely reflect changes in nutrient sources and primary producer composition associated with eddy-driven nutrient supply, as upwelled deep-water nitrate typically carries higher δ^15^N values and can lead to diatom blooms offshore (Casciotti et al., 2008; Rii et al., 2008). Despite these baseline shifts following the eddy, we still observed patterns characteristic of the IME in other seasons: higher δ^15^N_SAA_ values and lower δ^13^C Lys values in reef-associated assemblages compared to offshore surface assemblages. Integrating real-time oceanographic observations with isotopic sampling in future studies would improve the ability to distinguish transient mesoscale influences from persistent IME-driven nearshore–offshore gradients.

Given the pronounced nearshore–offshore gradients in zooplankton δ^13^C_EAA_, δ^15^N_SAA_, and TP values, an important remaining question is how far and when IME-derived signals persist offshore before pelagic baselines dominate consumer tissues. Our limited sampling at intermediate distances suggests that the offshore influence of the IME on consumers may be restricted in areas with steep bathymetry, where oceanic conditions develop within just a few km of shore. In contrast, regions with gentler bathymetry, like Kāneʻohe Bay on Oʻahu, may allow IME signals to extend farther offshore. The spatial extent and temporal persistence of the IME are likely shaped by multiple factors, including proximity to rivers, streams, and submarine groundwater discharge sites, human nutrient inputs (*e.g.*, sewage effluent or agricultural runoff), mesoscale oceanographic features (*e.g.*, eddies, internal waves, upwelling), and seasonal variation in rainfall and macronutrient availability (Hoover et al., 2006; Casciotti et al., 2008; Rii et al., 2008). These spatiotemporal dynamics may modify primary producer community composition and ultimately influence consumer δ^13^C_EAA_, δ^15^N_SAA_, and TP values. Accordingly, expanded sampling across intermediate offshore distances (1.5–20 km), sites spanning gradients in bathymetry, freshwater influence, and human activity, and multiple seasons is critical for characterizing the full spatial and temporal influence of the IME on near-island pelagic food webs. Further, offshore deep sampling in our contemporary sampling campaigns was not possible due to lack of access to a MOCNESS. In addition to increased sampling at intermediate offshore distances, future studies should also collect depth-discrete samples at all intermediate and offshore distances where possible to improve understanding of isotopic changes along nearshore–offshore and surface–deep gradients. Even so, our analyses demonstrate that spatial (reef *vs.* offshore) differences in amino acid isotopic values and trophic position estimates dominate over temporal variation across years, and that the observed patterns are not driven by seasonality.

Overall, combining δ^13^C_EAA_ and δ^15^N_SAA_ values into multivariate analysis allowed for clearer discrimination among reef, offshore surface, and offshore deep zooplankton in the Hawaiian Islands than either metric alone. Because these isotopic compositions are largely insensitive to fractionation during trophic transfer, this dual-isotope approach provides a method for tracking island-derived production through consumers in pelagic food webs. While the addition of TP metrics did not substantially improve classification in our dataset, they might be useful in other systems, and TDFs should be carefully considered in future applications that utilize TPs for assessment (McMahon & McCarthy, 2016; Shea, Drazen & Popp, 2025). Importantly, the choice of isotopic metrics used in LDAs may depend on the specific ecological context and analytical constraints of a given study. In cost-limited situations, combining TP metrics with δ^15^N_SAA_ values may be sufficient to discriminate coastal, pelagic surface, and pelagic deep food sources when δ^13^C_EAA_ analyses are not feasible. Conversely, LDAs incorporating δ^13^C_EAA_ values and TP metrics may provide a more biologically focused perspective on primary producer support and food web length while minimizing the influence of nitrogen source variability from δ^15^N_SAA_ values. Together, these complementary approaches highlight the potential flexibility of AA-CSIA-based multivariate frameworks for resolving ecological structure across biological and geochemical divisions.

Extending this dual-isotope framework to micronekton and pelagic predators could yield valuable insights into IME-driven habitat connectivity, particularly for slope-associated mesopelagic micronekton that migrate shoreward at night to exploit enhanced nearshore resources (Reid et al., 1991; Benoit-Bird, Zirbel & McManus, 2008). Determining whether these migrations are fueled by island-derived prey would clarify a critical energetic link between coastal and open-ocean ecosystems. Because micronekton form the forage base for culturally and economically important predators like tunas, billfishes, seabirds, and marine mammals (Skillman, 1998; West et al., 2009; Abecassis et al., 2015), tracing the propagation of IME-derived production to higher trophic levels carries direct implications for ecosystem management, conservation, and fisheries sustainability.

## Conclusions

This study demonstrates that the IME leaves distinct isotopic biogeochemical imprints on reef-associated zooplankton. By integrating δ^13^C_EAA_, δ^15^N_SAA_, and TP analyses, we reveal how these imprints differ between coastal and pelagic food webs. Nearshore–offshore isotopic gradients in Hawaiian zooplankton likely reflect shifts in phytoplankton community composition, nitrogen source utilization, and food web complexity, all of which are key processes that define the spatial extent of island productivity in oligotrophic regions. The observation of similar isotopic patterns in both Hawaiian and Maldivian systems suggests that IME-driven changes in phytoplankton along nutrient gradients may be a widespread feature of island ecosystems. The combined δ^13^C_EAA_ and δ^15^N_SAA_ framework developed here provides a conservative tracer for quantifying island-derived production and mapping ecological connectivity across oceanic habitats. As climate change expands oligotrophic gyres and intensifies stratification, understanding how island “oases” sustain pelagic food webs will be critical for predicting shifts in biodiversity, productivity, and resource availability. Extending these isotopic tracers to micronekton and their predators represents a crucial next step, with the potential to directly inform conservation strategies and sustainable fisheries management across the tropical Pacific and other regions worldwide.

## Supplemental Information

10.7717/peerj.21076/supp-1 Supplemental Information 1 Zooplankton collection information Distance from shore was calculated using distance to closest land mass with the measurement tool in Google Earth.

10.7717/peerj.21076/supp-2 Supplemental Information 2 Summary of linear regression results comparing Hawaiian zooplankton δ^13^C essential amino acid values with natural log-transformed distance from shore

10.7717/peerj.21076/supp-3 Supplemental Information 3 Summary of statistical test results comparing Hawaiian zooplankton δ^13^C essential amino acid values among reef, offshore surface, and offshore deep collection habitats

10.7717/peerj.21076/supp-4 Supplemental Information 4 Summary of statistical test results comparing Maldivian zooplanktivore δ^13^C essential amino acid values among presumed consumer diet groups (reef planktivores vs. pelagic planktivores)

10.7717/peerj.21076/supp-5 Supplemental Information 5 Summary of multivariate statistical test comparing Hawaiian zooplankton δ^13^C essential amino acid values across collection habitats

10.7717/peerj.21076/supp-6 Supplemental Information 6 Summary of the multivariate statistical analysis comparing primary producer δ^13^C essential amino acid values from Stahl, Rynearson & McMahon (2023)

10.7717/peerj.21076/supp-7 Supplemental Information 7 Summary of linear regression results comparing Hawaiian zooplankton δ^15^N source amino acid values with natural log-transformed distance from shore

10.7717/peerj.21076/supp-8 Supplemental Information 8 Summary of statistical test results comparing Hawaiian zooplankton δ^15^N source amino acid values among reef, offshore surface, and offshore deep collection habitats

10.7717/peerj.21076/supp-9 Supplemental Information 9 Summary of statistical test results for linear discriminant analysis comparing Hawaiian zooplankton δ^13^*C* essential amino acid and δ^15^N source amino acid values across collection habitats

10.7717/peerj.21076/supp-10 Supplemental Information 10 Summary of linear regression results comparing Hawaiian zooplankton TP estimates with natural log-transformed distance from shore

10.7717/peerj.21076/supp-11 Supplemental Information 11 Summary of statistical test results comparing Hawaiian zooplankton TP values among reef, offshore surface, and offshore deep collection habitats

10.7717/peerj.21076/supp-12 Supplemental Information 12 Summary of statistical test results for LDA comparing Hawaiian zooplankton δ^13^*C* essential amino acid, δ^15^*N* source amino acid values, and trophic position estimates across collection habitats

10.7717/peerj.21076/supp-13 Supplemental Information 13 Boxplots of mean-centered δ^13^C_EAA_ values (Leu, Lys, Phe, Thr, and Val) from Maldivian planktivores grouped by presumed diet, *i.e.* reef (*n* = 25) *vs.* pelagic (*n* = 8) plankton Data and presumed groupings are from Skinner et al. (2021). Horizontal bars represent significant comparisons, with ** denoting *p* < 0.01 and *** denoting *p* ¡ 0.001.

10.7717/peerj.21076/supp-14 Supplemental Information 14 LDA of Hawaiian zooplankton δ^13^C_EAA_ values (Ile, Leu, Lys, Phe, Thr, and Val) between reef, offshore surface, and offshore deep collection habitats Colored ellipses represent 95% confidence intervals around zooplankton groups.

10.7717/peerj.21076/supp-15 Supplemental Information 15 LDA of Hawaiian zooplankton mean-centered δ^13^C_EAA_ values, δ^15^N_SAA_ values, and TP estimates between reef, offshore surface, and offshore deep habitats Pairwise PERMANOVA revealed significant differences between all groups. Colored ellipses represent 95% confidence intervals around habitat groups.

## Additional Information and Declarations

### Data Availability

The following information was supplied regarding data availability: The raw data is available at Zenodo: Stoner-Osborne, B., Popp, B., García-Seoane, R., Wall, C., Drazen, J., & Wallsgrove, N. (2026). Data and Code for Publication: “Amino acid compound-specific isotope analysis reveals island mass effect subsidies in reef-associated Hawaiian zooplankton”. PeerJ. https://doi.org/10.5281/zenodo.18721290
