# Supplementary material for: Amino acid compound-specific isotope analysis reveals island mass effect subsidies in reef-associated Hawaiian zooplankton
Source: PeerJ. 2026 Apr 29;14:e21076. doi: 10.7717/peerj.21076 (PMC13135334; doi:10.7717/peerj.21076)
Supplement: Supplemental Information 1 — Distance from shore was calculated using distance to closest land mass with the measurement tool in Google Earth. [file peerj-14-21076-s001.docx]

| **Island** | **Location** | **Collection Season** | **Habitat** | **Distance from Shore** | **Maximum Tow Depth** | **Coordinates** |
| --- | --- | --- | --- | --- | --- | --- |
| Hawaiʻi | Kona Coast  (Old Kona Airport Beach Park) | Oct 2022 | Reef | <1 km | 3 m | 19°38'19"N 156°00'44"W |
|  |  |  | Offshore Surface | 20 km | 100 m | 19°38'04"N 156°12'15"W |
|  |  |  | Offshore Surface | 35 km | 100 m | 19°37'38"N 156°21'31"W |
|  |  |  | Offshore Surface | 50 km | 100 m | 19°37'25"N 156°28'43"W |
|  | Kona Coast  (Shoreline Park) | Dec 2023 | Reef | <1 km | 3 m | 19°27'22"N 155°55'29"W |
|  |  |  | Offshore Surface | 1.5 km | 25 m | 19°27'34"N 155°56'33"W |
|  |  |  | Offshore Surface | 3 km | 50 m | 19°27'09"N 155°57'28"W |
|  |  |  | Offshore Surface | 28 km | 100 m | 19°24'43"N 156°11'13"W |
| Oʻahu | Station ALOHA | Apr 2000 | Offshore Surface | 100 km | 160 m | 22°45'00"N 158°00'00"W |
|  |  | Aug 2000 |  |  | 160 m |  |
|  |  | Jan 2005 |  |  | 160 m |  |
|  |  | Aug 2011 | Offshore Surface  &  Offshore Deep | 100 km | 1,000 m | 22°45'00"N 158°00'00"W |
|  |  | Feb 2014 |  |  | 1,500 m |  |
|  |  | Aug 2014 |  |  | 1,500 m |  |
|  | Waiʻanae Coast  (Electric Beach) | Oct 2022 | Reef | <1 km | 3 m | 21°21'12"N 158°07'53"W |
|  | North Shore  (Sunset Beach) |  | Reef | <1 km | 3 m | 21°39'23"N 158°03'44"W |
|  | Waimanalo  (Kaiona Beach Park) |  | Reef | <1 km | 3 m | 21°19'35"N 157°40'55"W |
