# Supplementary material for: Amino acid compound-specific isotope analysis reveals island mass effect subsidies in reef-associated Hawaiian zooplankton
Source: PeerJ. 2026 Apr 29;14:e21076. doi: 10.7717/peerj.21076 (PMC13135334; doi:10.7717/peerj.21076)
Supplement: Supplemental Information 2 [file peerj-14-21076-s002.docx]

| **Essential Amino Acid** | **Test Type** | **Statistic** | **Degrees of Freedom** | **Regression Equation** | **Adjusted R^2^** | **Adjusted *p*-values** |
| --- | --- | --- | --- | --- | --- | --- |
| Ile | *t*-test | t = -0.793 | 24 | y = - 0.0737x + 2.24 | -0.015 | 0.436 |
| Leu | *t*-test | t = -0.414 | 24 | y = - 0.0237x - 4.95 | -0.034 | 0.683 |
| Lys | *t*-test | t = 3.420 | 24 | y = 0.419x + 1.41 | 0.300 | 0.002 |
| Phe | *t*-test | t = -3.227 | 24 | y = - 0.307x - 3.7 | 0.274 | 0.004 |
| Thr | *t*-test | t = 3.083 | 24 | y = 0.259x + 6.61 | 0.254 | 0.005 |
| Val | *t*-test | t = -3.097 | 24 | y = - 0.274x - 1.61 | 0.256 | 0.005 |
