# Supplementary material for: Amino acid compound-specific isotope analysis reveals island mass effect subsidies in reef-associated Hawaiian zooplankton
Source: PeerJ. 2026 Apr 29;14:e21076. doi: 10.7717/peerj.21076 (PMC13135334; doi:10.7717/peerj.21076)
Supplement: Supplemental Information 3 [file peerj-14-21076-s003.docx]

| **Essential Amino Acid** | **Test Type** | **Statistic** | **Degrees of Freedom** | **Adjusted *p*-value** | **Effect Size** | **Pairwise Comparisons (post-hoc)** | **Pairwise *p*-value** |
| --- | --- | --- | --- | --- | --- | --- | --- |
| Ile | ANOVA | F = 0.640 | 2, 23 | 0.537 | η^2^ = 0.053 | — | — |
| Leu | ANOVA | F = 0.208 | 2, 23 | 0.814 | η^2^ = 0.018 | — | — |
| Lys | Kruskal-Wallis | H = 5.986 | 2 | 0.050 | ε^2^ = 0.173 | — | — |
| Phe | ANOVA | F = 5.061 | 2, 23 | 0.015 | η^2^ = 0.306 | Reef vs. Offshore Surface | 0.194 |
|  |  |  |  |  |  | Reef vs. Offshore Deep | 0.012 |
|  |  |  |  |  |  | Offshore Surface vs. Offshore Deep | 0.150 |
| Thr | ANOVA | F = 6.733 | 2, 23 | 0.005 | η^2^ = 0.369 | Reef vs. Offshore Surface | 0.186 |
|  |  |  |  |  |  | Reef vs. Offshore Deep | 0.004 |
|  |  |  |  |  |  | Offshore Surface vs. Offshore Deep | 0.054 |
| Val | Kruskal-Wallis | H = 5.901 | 2 | 0.052 | ε^2^ = 0.170 | — | — |
