# Supplementary material for: Amino acid compound-specific isotope analysis reveals island mass effect subsidies in reef-associated Hawaiian zooplankton
Source: PeerJ. 2026 Apr 29;14:e21076. doi: 10.7717/peerj.21076 (PMC13135334; doi:10.7717/peerj.21076)
Supplement: Supplemental Information 4 [file peerj-14-21076-s004.docx]

| **Essential Amino Acid** | **Test Type** | **Statistic** | **Degrees of Freedom** | **Adjusted *p*-value** | **Effect Size** |
| --- | --- | --- | --- | --- | --- |
| Leu | Welch’s *t*-test | t = -3.751 | 23.804 | 0.002 | d = -1.277 |
| Lys | Welch’s *t*-test | t = 3.676 | 10.240 | 0.004 | d = 1.565 |
| Phe | Mann-Whitney U test | W = 0 | - | < 0.001 | r = 0.731 |
| Thr | Mann-Whitney U test | W = 200 | - | < 0.001 | r = 0.731 |
| Val | Mann-Whitney U test | W = 122 | - | 0.374 | r = 0.161 |
