# Supplementary material for: Amino acid compound-specific isotope analysis reveals island mass effect subsidies in reef-associated Hawaiian zooplankton
Source: PeerJ. 2026 Apr 29;14:e21076. doi: 10.7717/peerj.21076 (PMC13135334; doi:10.7717/peerj.21076)
Supplement: Supplemental Information 6 [file peerj-14-21076-s006.docx]

| **Test Type** | **Statistic** | **Degrees of Freedom** | **Adjusted *p*-value** | **Effect Size** | **Pairwise Comparisons (post-hoc)** | **Pairwise *p*-value** |
| --- | --- | --- | --- | --- | --- | --- |
| PERMANOVA | F = 41.967 | 3, 65 | 0.001 | R^2^ = 0.660 | Diatom vs. Dinoflagellate | 0.001 |
|  |  |  |  |  | Diatom vs. Prasinophyte | 0.001 |
|  |  |  |  |  | Diatom vs. Raphidophyte | 0.001 |
|  |  |  |  |  | Dinoflagellate vs. Prasinophyte | 0.001 |
|  |  |  |  |  | Dinoflagellate vs. Raphidophyte | 0.001 |
|  |  |  |  |  | Prasinophyte vs. Raphidophyte | 0.001 |
