# Supplementary material for: Amino acid compound-specific isotope analysis reveals island mass effect subsidies in reef-associated Hawaiian zooplankton
Source: PeerJ. 2026 Apr 29;14:e21076. doi: 10.7717/peerj.21076 (PMC13135334; doi:10.7717/peerj.21076)
Supplement: Supplemental Information 7 [file peerj-14-21076-s007.docx]

| **Source Amino Acid** | **Test Type** | **Statistic** | **Degrees of Freedom** | **Regression Equation** | **Adjusted R^2^** | **Adjusted *p*-values** |
| --- | --- | --- | --- | --- | --- | --- |
| Lys | *t*-test | t = -6.172 | 54 | y = - 0.445x + 0.797 | 0.403 | < 0.001 |
| Phe | *t*-test | t = -13.435 | 57 | y = - 0.936x + 1.38 | 0.756 | < 0.001 |
