# Supplementary material for: Amino acid compound-specific isotope analysis reveals island mass effect subsidies in reef-associated Hawaiian zooplankton
Source: PeerJ. 2026 Apr 29;14:e21076. doi: 10.7717/peerj.21076 (PMC13135334; doi:10.7717/peerj.21076)
Supplement: Supplemental Information 8 [file peerj-14-21076-s008.docx]

| **Source Amino Acid** | **Test Type** | **Statistic** | **Degrees of Freedom** | **Adjusted *p*-value** | **Effect Size** | **Pairwise Comparisons (post-hoc)** | **Pairwise *p*-value** |
| --- | --- | --- | --- | --- | --- | --- | --- |
| Lys | Kruskal-Wallis | H = 56.168 | 2 | < 0.001 | ε^2^ = 0.463 | Reef vs. Offshore Surface | < 0.001 |
|  |  |  |  |  |  | Reef vs. Offshore Deep | 0.847 |
|  |  |  |  |  |  | Offshore Surface vs. Offshore Deep | < 0.001 |
| Phe | Kruskal-Wallis | H = 57.540 | 2 | < 0.001 | ε^2^ = 0.306 | Reef vs. Offshore Surface | < 0.001 |
|  |  |  |  |  |  | Reef vs. Offshore Deep | 0.006 |
|  |  |  |  |  |  | Offshore Surface vs. Offshore Deep | < 0.001 |
