# Supplementary material for: Amino acid compound-specific isotope analysis reveals island mass effect subsidies in reef-associated Hawaiian zooplankton
Source: PeerJ. 2026 Apr 29;14:e21076. doi: 10.7717/peerj.21076 (PMC13135334; doi:10.7717/peerj.21076)
Supplement: Supplemental Information 10 [file peerj-14-21076-s010.docx]

| **TP Estimate** | **Test Type** | **Statistic** | **Degrees of Freedom** | **Regression Equation** | **Adjusted R^2^** | **Adjusted *p*-values** |
| --- | --- | --- | --- | --- | --- | --- |
| TP_(Ala-Phe)_ | *t*-test | t = 6.262 | 116 | y = 0.23x + 3.02 | 0.246 | < 0.001 |
| TP_(Glx-Phe)_ | *t*-test | t = 7.095 | 116 | y = 0.14x + 2.03 | 0.297 | < 0.001 |
