# Supplementary figures and images for: Amino acid compound-specific isotope analysis reveals island mass effect subsidies in reef-associated Hawaiian zooplankton
Source: PeerJ. 2026 Apr 29;14:e21076. doi: 10.7717/peerj.21076 (PMC13135334; doi:10.7717/peerj.21076)

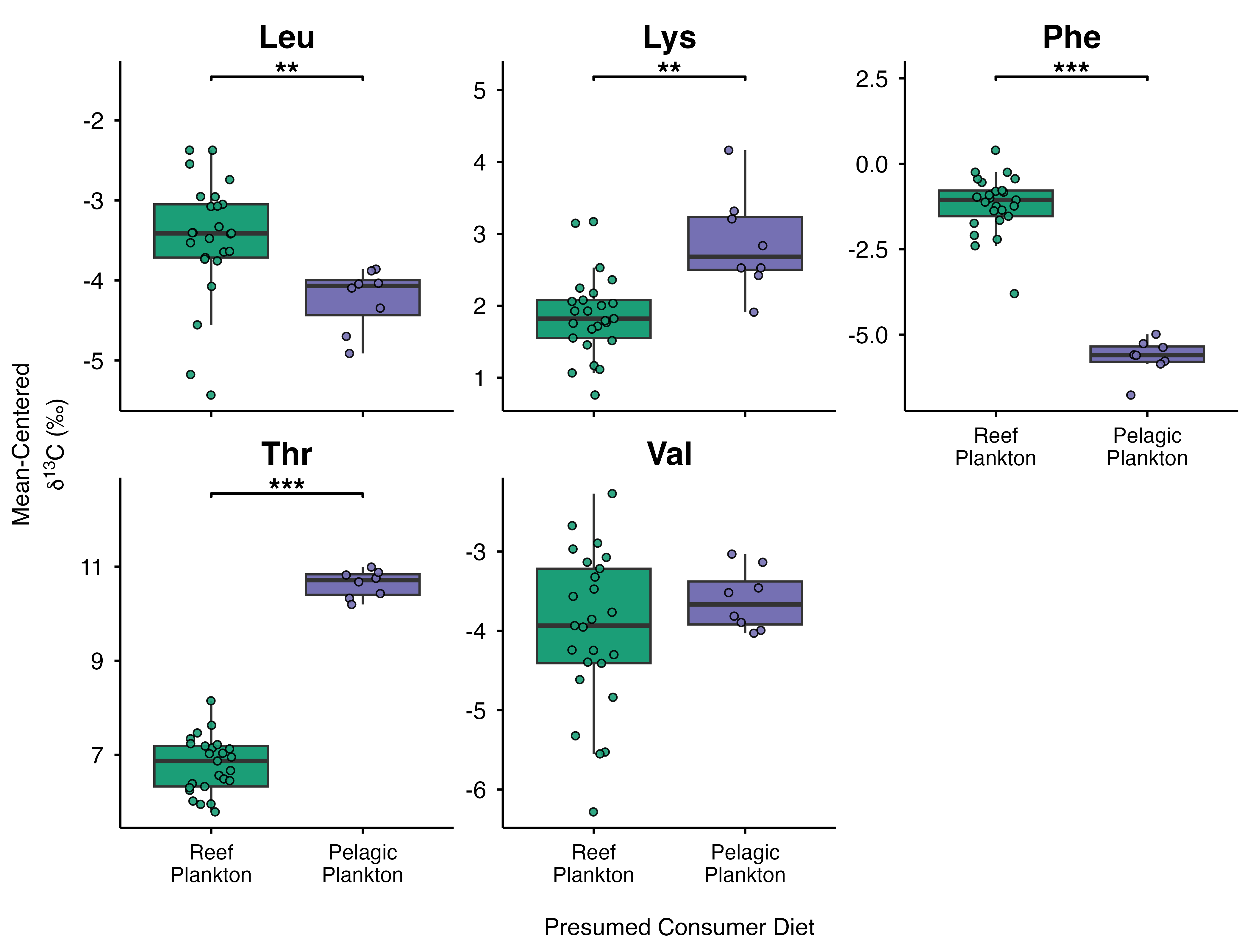

Supplement: Supplemental Information 13 — Data and presumed groupings are from Skinner et al. (2021). Horizontal bars represent significant comparisons, with ** denoting p < 0.01 and *** denoting p ¡ 0.001. [file peerj-14-21076-s013.png]

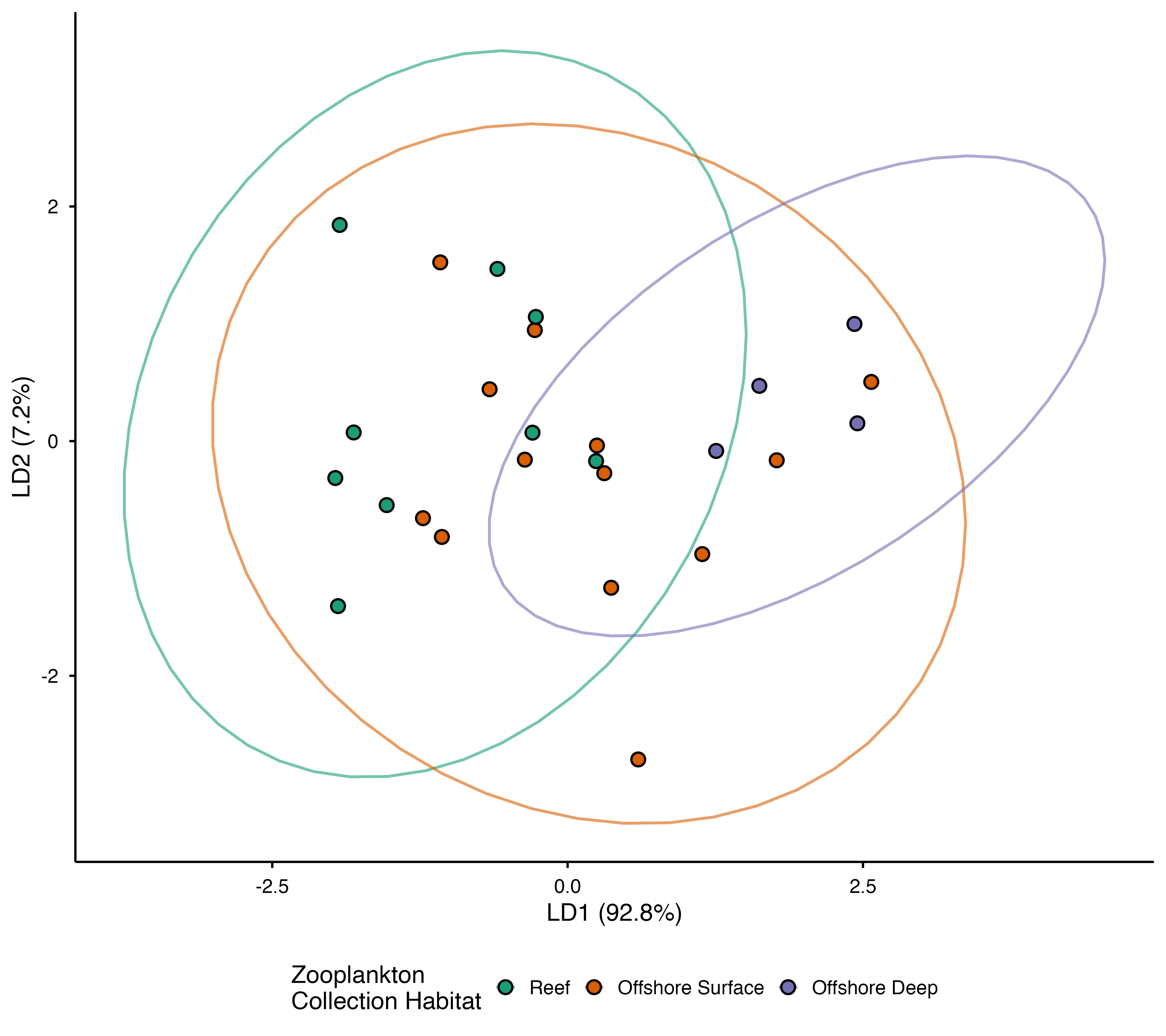

Supplement: Supplemental Information 14 — Colored ellipses represent 95% confidence intervals around zooplankton groups. [file peerj-14-21076-s014.png]

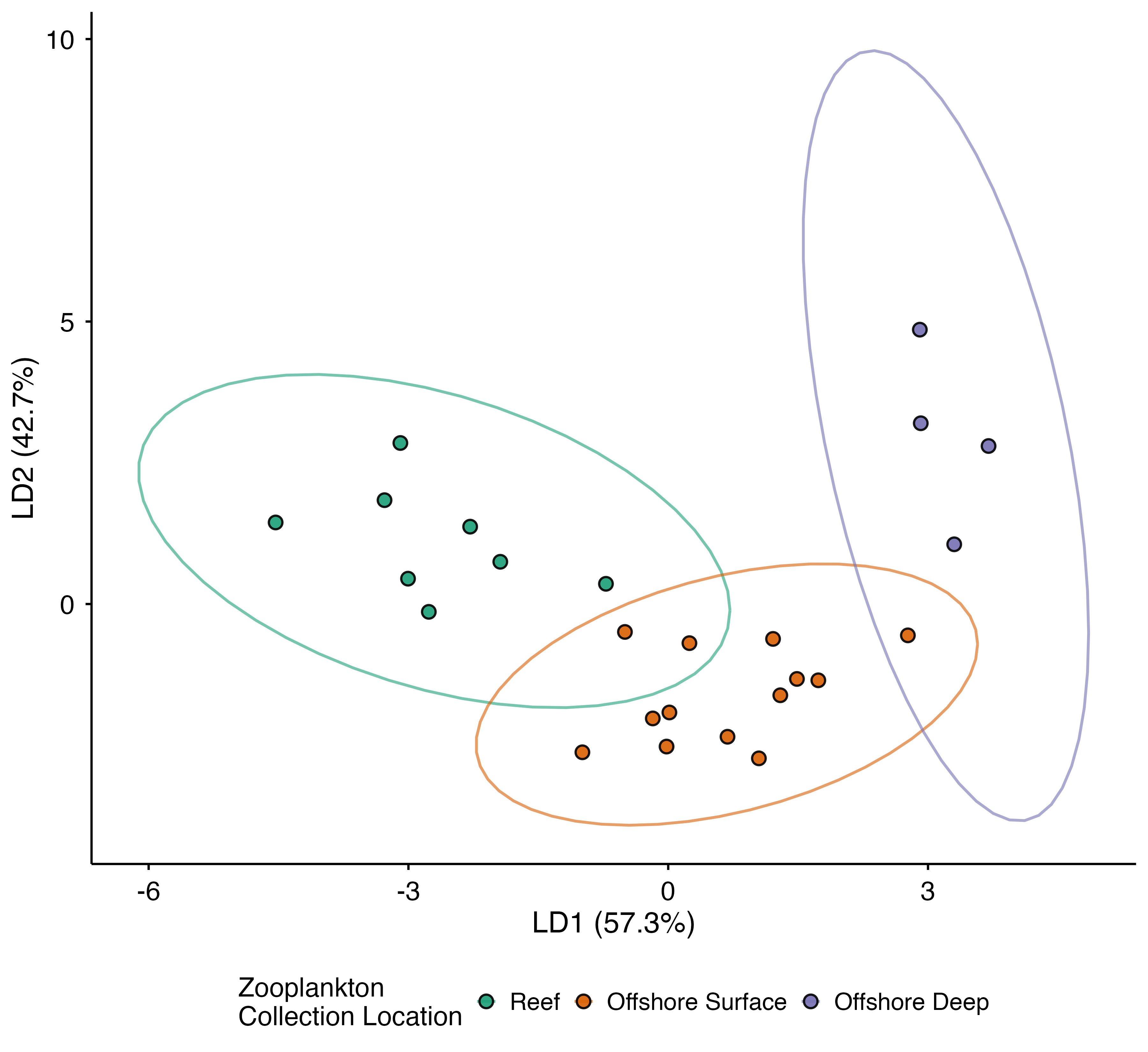

Supplement: Supplemental Information 15 — Pairwise PERMANOVA revealed significant differences between all groups. Colored ellipses represent 95% confidence intervals around habitat groups. [file peerj-14-21076-s015.png]
